# Proposed Allosteric Inhibition of Cyclin-Dependent Kinase 4 by a Proline-Derived Acylsemicarbazide Compound with Antiproliferative Activity in Breast Cancer Cells

**DOI:** 10.3390/ph19081166

**Published:** 2026-07-26

**Authors:** Xu Huang, Qingyang Nian, Xizhe Sun, Yuheng Zhou, Yuxin Wang, Jiayin Yue, Fanhao Meng, Jingwei Liang

**Affiliations:** 1 Engineering Research Center of Tropical Medicine Innovation and Transformation of Ministry of Education, School of Pharmacy, Hainan Medical University, Haikou 571199, China; 17779273961@163.com (X.H.); qq294081580@163.com (Q.N.); hy0308028@muhn.edu.cn (X.S.); qw1761311640@163.com (Y.Z.); 15708987336@163.com (Y.W.); 2 School of Pharmacy, China Medical University, Shenyang 110122, China; 3 School of Pharmacy, Shenyang Medical University, Shenyang 110034, China

**Keywords:** CDK4, molecular dynamics simulations, Markov state model, allostery, allosteric inhibitor

## Abstract

**Background:** Cyclin-dependent kinase 4 (CDK4) is a key regulator of cell-cycle progression and an established therapeutic target for breast cancer. Although the unique architecture of its ATP-binding site has enabled the development of highly selective inhibitors, the emergence of acquired resistance highlights the need for alternative therapeutic strategies targeting protein conformational regulation. **Methods:** The conformational landscape of CDK4 was investigated in this study using accelerated molecular dynamics (aMD) simulations combined with Markov state model (MSM) analysis to identify cryptic conformational states and potential allosteric binding sites. Particular attention was given to the glycine-rich loop (G-loop), a critical structural element that shapes the ATP-binding pocket, and to the effects of compound **8i** (1-(2-(3-chlorobenzoyl)hydrazine-1- carbonyl)-N-(pyridin-3-yl)pyrrolidine-2-carboxamide), previously synthesized in our laboratory, on CDK4 dynamics. **Results:** A distinct conformational transition was identified in which the G-loop shifted toward the N-terminus, resulting in the exposure of a previously unrecognized allosteric pocket adjacent to the catalytic site. Compound 8i interacted with Leu147 and was associated with stabilization of conformational states that favor exposure of the cryptic pocket. **Conclusions:** These observations suggest that ligand binding may modulate the conformational landscape of CDK4 and favor formation of a cryptic pocket with potential allosteric characteristics. The identified conformational mechanism provides new insights into the dynamic regulation of CDK4 and suggests that stabilization of transient allosteric states represents a promising strategy for the rational design of next-generation CDK4 inhibitors with the potential to overcome resistance in breast cancer therapy.

## 1. Introduction

Cyclin-dependent kinases (CDKs) constitute a class of serine/threonine protein kinases that collaborate with cyclins to orchestrate and modulate the progression of the cell cycle at different phases. Their pivotal role in the regulation of the cell cycle has been well established [1]. Within tumor cells, inhibiting CDK activity instigates cell cycle arrest, impeding cell proliferation, thereby underscoring their significance as prime anti-tumor targets [2,3,4]. Notably, CDK2 emerges as a cyclin-dependent kinase pivotal for steering cells through the G1 phase and transitioning into the S phase. CDK2’s interaction with and activation by cyclin E sustains the phosphorylation of the retinoblastoma protein (RB) during the latter part of G1, facilitating the seamless traversal from G1 to S phase [5,6]. In the realm of anti-breast cancer drug research and development, inhibitors directed against CDK2 have garnered considerable attention. Several compounds have advanced to clinical studies, revealing promising anti-tumor efficacy [7]. Nevertheless, the potential adverse effects of these inhibitors observed in clinical trials cannot be disregarded, encompassing conditions such as neutropenia, leukopenia, and myelosuppression. These effects primarily stem from CDK2’s extensive substrate spectrum and its involvement in diverse signaling pathways. Excessive inhibition poses a substantial risk of disrupting normal cellular functions, thus challenging the progress of drug development for this specific target [8].

In recent years, CDK4/6 inhibitors represented by Palbociclib have been approved for the treatment of breast cancer. Compared with CDK2 inhibitors, CDK4/6 inhibitors also inhibit the proliferation of breast cancer cells by inducing G1/S phase arrest. But the difference is that CDK4/6 inhibitors do not affect other CDKs involved in the regulation of normal cell cycle, effectively reduce the side effects. It brought these inhibitors become the most valuable and promising anti-breast cancer drugs [9,10]. However, with the clinical application of CDK4/6 inhibitors, the number of drug-resistant cases is increasing, which seriously limits the effectiveness of these inhibitors [11,12].

Research findings have underscored the significant role of compensatory CDK4/6 activation in mediating breast cancer resistance to CDK2 inhibitors. A pivotal requisite for cells transitioning from G1 to S phase is the phosphorylation of the retinoblastoma protein (RB). Within the CDK family, CDK2 and CDK4 act in concert to intricately orchestrate the cell cycle progression from G1 to S phase, thereby collectively facilitating the RB protein’s phosphorylation process. In cases where CDK4 in breast cancer cells faces inhibition, unabated CDK2 activity persists, leading to the continued phosphorylation of the RB protein as a bypass pathway and the uninterrupted propulsion of cells into the S phase. Consequently, this phenomenon engenders drug resistance in breast cancer cells. To circumvent this challenge, concurrent inhibition of CDK4 and CDK2 has emerged as a potential solution to surmount drug resistance associated with CDK4/6 inhibitors [13]. However, the central hurdle in the development of dual-target inhibitors targeting CDK2 and CDK4 resides in the pronounced spatial divergence characterizing the active pockets of these two kinases [14]. This divergence predominantly stems from the conformational disparities exhibited by the G-loop domains implicated in the constitution of these active pockets.

In recent years, with the rapid development of computer technology, the combination of molecular dynamics (MD) simulation [15] and Markov state model (MSM) analysis [16] has been successfully applied to the study of protein folding [17], molecular recognition [18], and conformational dynamics [19,20]. Inspired by our previous work, we synthesized a novel CDK4 inhibitor designated as **8i** ((S)-1-(2-(3-chlorobenzoyl)hydrazine-1-carbonyl)-*N*-(pyridin-3-yl)pyrrolidine-2-carboxamide) in this study [21]. Combined with molecular dynamics and the MSM, we found a new allosteric mechanism in CDK4 and found an amino acid residue that is in the active pocket that contributes greatly to the stability of protein-ligand binding. This work provides a theoretical basis and new ideas for solving the problem of drug resistance of CDK4 inhibitors and designing new CDK2/4 dual-target inhibitors.

## 2. Result

### 2.1. Putative Allosteric Role of Leu147 in CDK4

**Figure 1:**
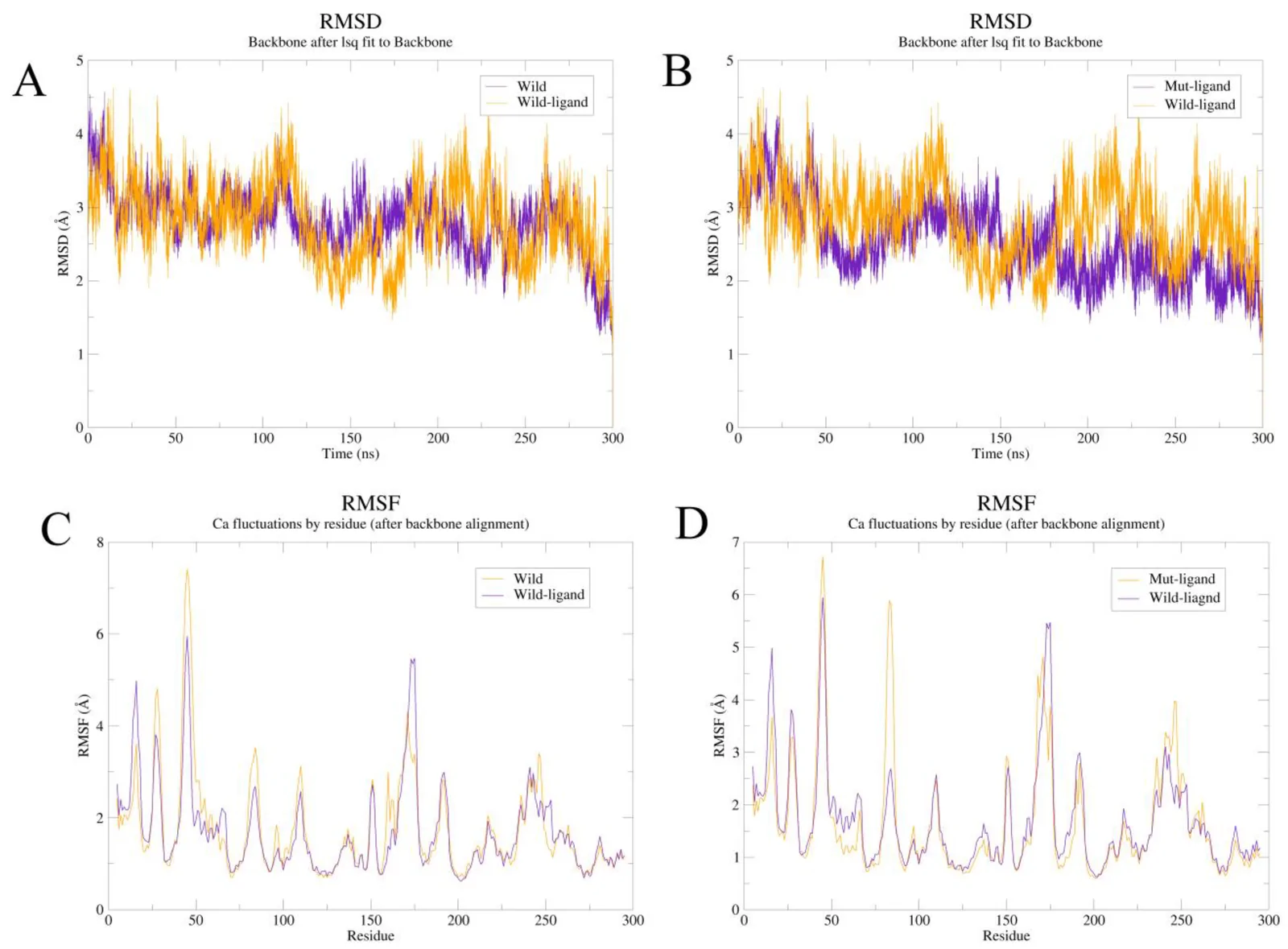
Molecular Dynamics Data of Wild-Type and Mutant CDK4. (**A**) Time evolution of RMSD showing that small-molecule **8i** binding accelerates structural stabilization of the wild-type CDK4 compared to the apo form; lower and faster-converging RMSD in the complex indicates a more compact, ligand-stabilized conformation. (**B**) Time evolution of RMSD comparing the wild-type CDK4–**8i** complex with the CDK4L147A mutant–**8i** complex, illustrating that the Leu147Ala mutation attenuates the ligand-induced conformational dynamics observed in the wild-type protein. (**C**) Per-residue RMSF profiles for the wild-type apo CDK4 and the wild-type CDK4–**8i** complex; reduced fluctuations in the complex reflect ligand-mediated stabilization of key structural regions, particularly around the G-loop. (**D**) Per-residue RMSF profiles comparing the wild-type CDK4–**8i** complex with the CDK4L147A mutant–**8i** complex; the altered fluctuation pattern in the mutant highlights the critical role of Leu147 in maintaining the allosteric behavior of CDK4 upon ligand binding.

**Figure 2:**
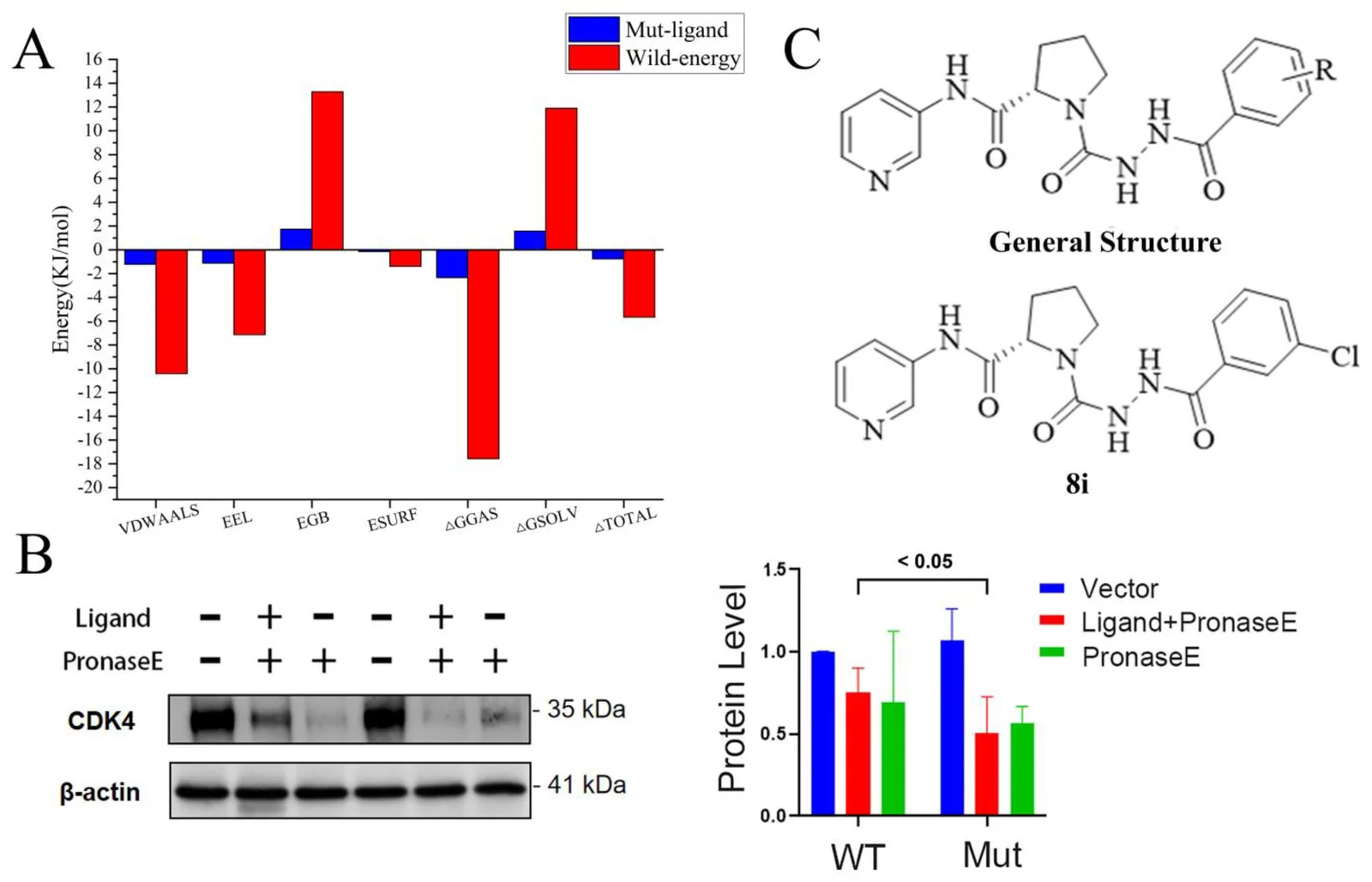
Comparison of CDK4 wild-type and mutant. (**A**) MM/GBSA binding free energy of compound **8i** with wild-type CDK4 and the CDK4^L147A^ mutant, demonstrating that the Leu147Ala substitution substantially reduces ligand binding affinity (values in kcal/mol; error bars represent standard deviation from three independent calculations). (**B**) Drug Affinity Responsive Target Stability (DARTS) assay monitored by Western blotting. CDK4 wild-type treated with **8i** shows markedly higher proteolytic resistance to Pronase E compared to the DMSO control, whereas the CDK4^L147A^ mutant fails to exhibit equivalent protection, confirming the dependence of ligand-induced stabilization on Leu147. (*n* = 3 independent experiments, Shown below) (**C**) Structural comparison between compound **8i** (1-(2-(3-chlorobenzoyl)hydrazine-1-carbonyl)-N-(pyridin-3-yl) pyrrolidine-2-carboxamide) and the general structure.

Inspired by our previous work [21], we synthesized several chiral enantiomers to further explore this skeleton. One of them is **8i**, which is an enantiomer of the previously reported compound **7i**. We found that its selectivity was different from the compounds we previously reported. Its inhibitory activity against CDK4 was similar to that against CDK2, while it had almost no inhibitory activity against CDK6 (Table 1). This indicates that it has unique combining characteristics. To further elucidate its interaction profile, we conducted a 300 ns accelerated molecular dynamics simulation of the protein–ligand complex (Figure 1A,B). Post-simulation analysis revealed that both the ligand and protein backbone exhibited RMSD (Supplementary Figure S2 for RMSD profiles) fluctuations of approximately 0.2 nm, indicating a stable system. The RMSD profiles over time from accelerated molecular dynamics simulations of the wild-type protein and the wild-type protein–ligand complex suggests that the small molecule **8i** can markedly accelerate conformational changes of the protein. Furthermore, analysis of the RMSD of **8i** in complex with both the wild-type and mutant proteins indicates that Leu147 may play an important role in the ligand-associated conformational regulation of the protein. In parallel, RMSF analysis was conducted for both the wild-type protein–ligand complex and the mutant protein–ligand complex (Figure 1C,D). RMSF analysis shows that binding of the small-molecule ligand is associated with conformational stabilization of wild-type CDK4, stabilizing key regions and reducing residue flexibility. The Leu147 to Ala147 mutation significantly alters local dynamics, changing fluctuations in specific areas. These results suggest that ligand binding alters CDK4 conformational dynamics and that Leu147 contributes to maintaining these ligand-associated structural changes. A representative 50 ns trajectory was extracted near the end of the accelerated molecular dynamics, and the binding free energy was calculated using the single-trajectory method in gmx MMPBSA [22], yielding a value of −5.67 kcal/mol (Figure 2A).

The CDK4^L147A^ mutant was constructed using Modeller 10.2. Following energy minimization, the protein model was successfully validated using PROCHECK and a 300 ns molecular dynamics simulation of the mutant–small molecule complex was performed. Comparative analysis with the CDK4 wild-type revealed that the protein backbone RMSD of the CDK4^L147A^ mutant fluctuated around 0.3 nm within a 0.1 nm range. The RMSD of the wild-type protein–ligand complex changed more rapidly over time than that of the mutant complex, suggesting that the small-molecule ligand **8i** significantly accelerates the conformational dynamics of the protein, while Leu147 appears to contribute to ligand-associated conformational regulation. To corroborate the findings above, we conducted Drug Affinity Target Stability (DARTS) experiments on both wild type and mutant CDK4 proteins, analyzing the samples through Western blot (WB). When bound with the small molecule **8i**, the wild-type CDK4’s stability significantly increased, it resisted to enzymatic hydrolysis by Pronase E. Conversely, due to the Leu147 mutation, the CDK4^L147A^ protein failed to bind with **8i**. It exhibited reduced binding stability with small molecules, resulting in a weakened resistance to enzymatic hydrolysis (Figure 2B). Furthermore, to elucidate the rational design concept that led to the discovery of **8i**, a structural comparison among the general structure from our previous study, and compound **8i** is presented in Figure 2C [21].

**Figure 3:**
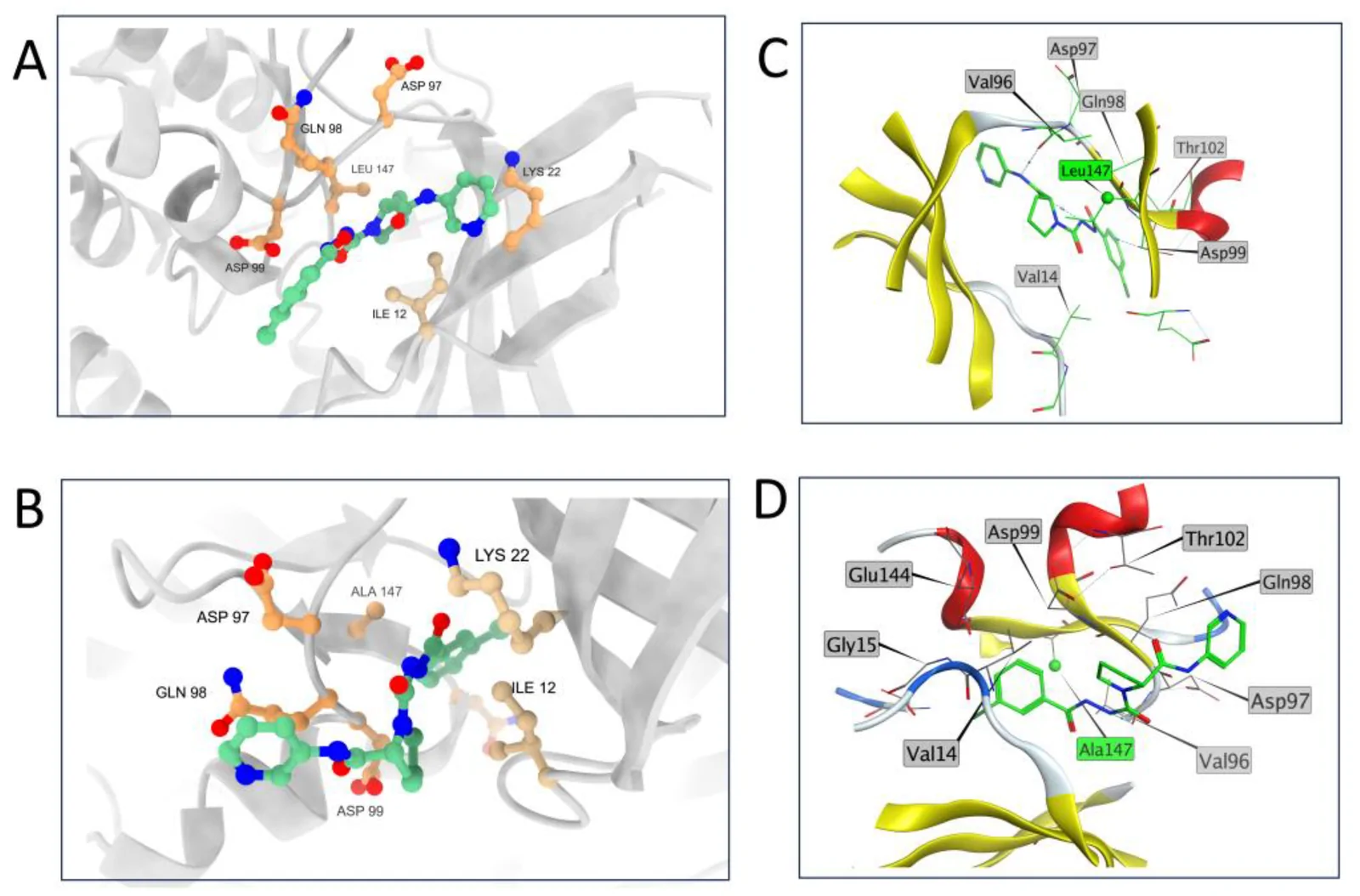
Three-dimensional interaction diagram of compound **8i** bound to CDK4. The ligand (compound **8i**) is shown as a green stick representation throughout all panels. (**A**) Cartoon representation of the wild-type CDK4–**8i** complex showing the ligand deeply embedded within a compact, well-defined binding pocket; surrounding residues tightly enclose **8i**, forming a stable hydrophobic enclosure. (**B**) Cartoon representation of the CDK4L147A mutant–**8i** complex revealing a noticeably enlarged and open pocket in which **8i** exhibits a positional shift and partial solvent exposure, indicative of reduced binding stability. (**C**) Close-up surface representation of the wild-type binding pocket, highlighting the steric and hydrophobic contributions of Leu147 to pocket geometry and ligand confinement. (**D**) Corresponding surface view of the CDK4L147A mutant pocket, demonstrating that loss of the leucine side chain creates excess cavity volume that compromises the geometric closure required for high-affinity binding.

In the wild-type CDK4, the ligand **8i** adopts a deeply inserted binding conformation, stably embedded within the protein’s binding pocket (Figure 3A). The surrounding residues form a well-defined enclosure, tightly wrapping the ligand and resulting in a compact and stable structure. In contrast, in the L147A mutant, the **8i** ligand exhibits a noticeable positional shift, with incomplete embedding and partial exposure to the solvent, leading to a looser and less confined binding pocket (Figure 3B). Leucine 147, a moderately sized, hydrophobic residue, is located at the edge or semi-enclosed surface of the binding pocket. It plays a crucial role by providing steric support or compression to constrain the ligand’s position, forming a hydrophobic platform that promotes π–hydrophobic interactions or clustering, and maintaining the geometric closure of the pocket to prevent ligand slippage. When mutated to alanine, which has a smaller side chain and lower hydrophobicity, the pocket becomes enlarged and lacks effective spatial filling, resulting in greater conformational freedom but reduced stability of the ligand (Figure 3C,D).

**Figure 4:**
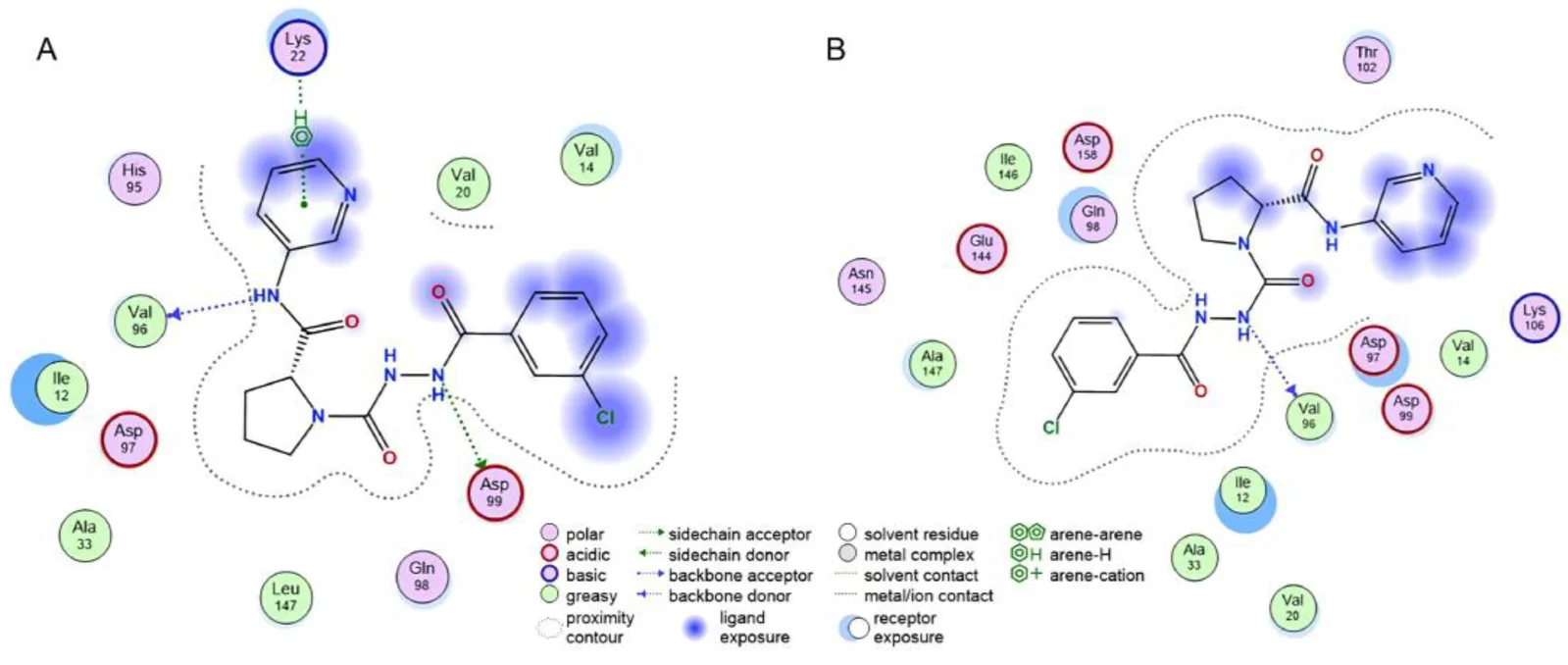
2D ligand–protein interaction diagram of compound **8i** bound to CDK4, generated using MOE 2022. (**A**) Interaction map of compound **8i** with wild-type CDK4 showing a cooperative binding network in which the ligand is stabilized by multiple hydrogen-bonding and polar interactions together with favorable contacts within a hydrophobic pocket surrounding Leu147. (**B**) Interaction map of compound **8i** with the CDK4^L147A^ mutant showing that substitution of Leu147 with alanine disrupts the local interaction network, resulting in ligand displacement, loss of key hydrogen-bonding interactions, and increased solvent exposure, consistent with reduced binding stability. Residues are color-coded according to their physicochemical properties (green, hydrophobic; red, acidic; blue, basic; and purple, polar). Blue dashed lines indicate hydrogen-bonding interactions, green dashed lines indicate arene–H and arene–cation interactions, the gray dotted contour denotes the receptor proximity contour, and blue and gray halos represent ligand and receptor exposure, respectively.

Ligand interaction diagrams further reveal interaction changes. In the wild-type CDK4, **8i** forms hydrophobic interactions (indicated by yellow/green lines) near Leu147, potentially involving van der Waals contact surfaces (Figure 4A). Additionally, it forms one to three hydrogen bonds or polar interactions (blue lines) with surrounding residues, creating a multipoint cooperative interaction network that stabilizes ligand positioning. In contrast, in the L147A mutant, the loss of hydrophobic packing with the original Leu147 residue leads to a local misalignment of the ligand, resulting in the loss of other hydrogen bonding interactions (Figure 4B). The overall interaction network is reduced and more unevenly distributed, leading to significantly weakened binding affinity. Although Leu147 may not directly form hydrogen bonds, its spatial conformation and hydrophobic nature contribute to a favorable “hydrophobic platform” and help maintain the positioning of nearby polar residues, thereby indirectly stabilizing other hydrogen bonds. This site thus plays both a structural support and an indirect enhancing role in ligand binding.

Structural analysis showed that Leu147 does not form identifiable hydrogen bonds, salt bridges, or π-stacking interactions with **8i**. However, its proximity to the ligand and the observed conformational changes in surrounding residues during the simulations suggest that Leu147 may contribute to local structural rearrangements associated with ligand binding. Based on these computational observations, we propose that Leu147 could function as a conformational modulator within the binding pocket. Further experimental studies will be required to validate this proposed role. One possible explanation consistent with our simulation results is that ligand binding may induce subtle steric effects around Leu147, leading to local side-chain rearrangements and changes in the geometry of neighboring residues. Such structural adjustments could contribute to formation of a binding environment more favorable for ligand accommodation. We emphasize that this model represents a computational hypothesis derived from molecular dynamics analyses and has not yet been experimentally verified.

A potential interpretation of the simulation data is that ligand binding may alter the conformational preferences of the local structural environment surrounding Leu147. Such local changes could, in principle, influence more distant regions of the protein through coupled structural motions. Within this computational framework, Leu147 may represent one component of a broader network of residues associated with conformational regulation. However, the existence of specific signal propagation pathways, dynamic coupling networks, or long-range allosteric communication was not directly tested in the present study and therefore should be regarded as a hypothesis requiring further experimental validation.

**Figure 5:**
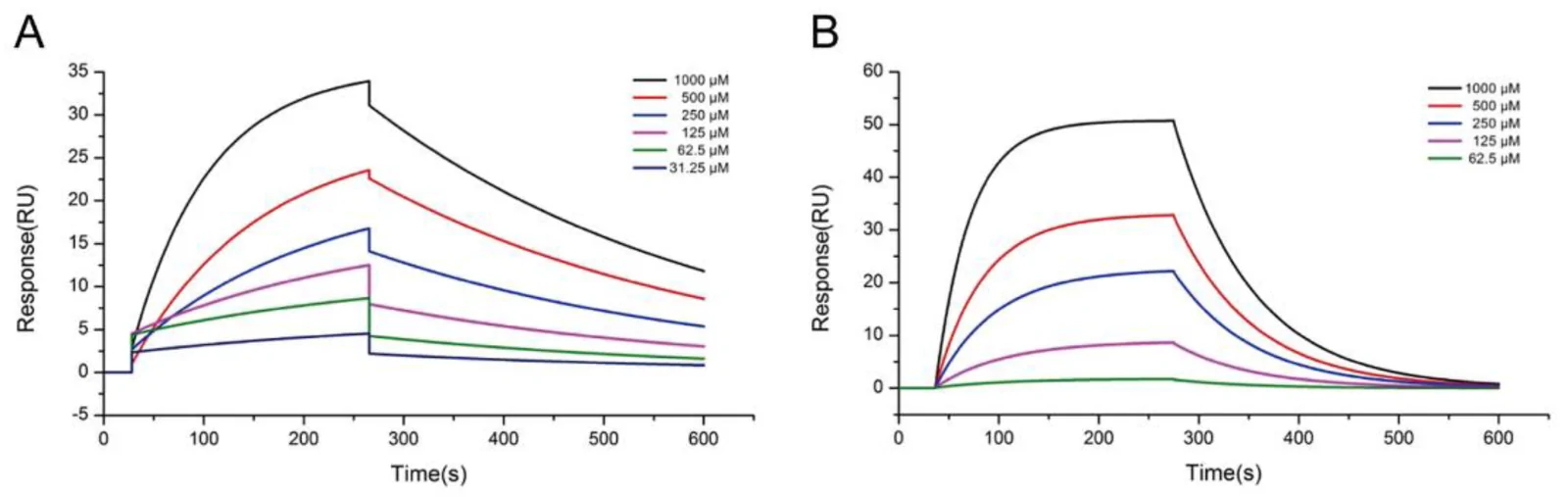
Surface Plasmon Resonance (SPR) analysis of **8i** binding to CDK4. (**A**) Sensorgram and steady-state fitting curve for **8i** binding to wild-type CDK4 (concentration range: 31.25–1000 μmol/L); the derived equilibrium dissociation constant (Kd) is 0.289 mmol/L, indicating moderate binding affinity. (**B**) Sensorgram and steady-state fitting curve for **8i** binding to the CDK4^L147A^ mutant (concentration range: 62.5–1000 μmol/L); the Kd increases to 0.774 mmol/L, demonstrating that the Leu147Ala mutation substantially reduces binding affinity and con-firming the critical role of Leu147 in mediating the compound-protein interaction. RU, response units.

To validate the binding affinity of **8i** to wild type CDK4 and mutant CDK4 respectively, we conducted Surface Plasmon Resonance (SPR) experiments (Figure 5). The proteins were immobilized on a Sensor Chip teem with -COOH, **8i** was tested at concentrations ranging from 31.25 to 1000 μmol/L for wild type and 62.5 to 1000 μmol/L for the mutant respectively. The sensorgram fitting curve indicated a Kd value of 0.289 mmol/L for CDK4 wild type with **8i** and 0.774 mmol/L for the CDK4 mutant with **8i**. These results underscored the higher affinity of CDK4 wild type for **8i** compared to the mutant, highlighting the impact of the Leu147 mutation on the binding capability of proteins and small molecules.

### 2.2. Putative Allosteric Process of CDK4

**Figure 6:**
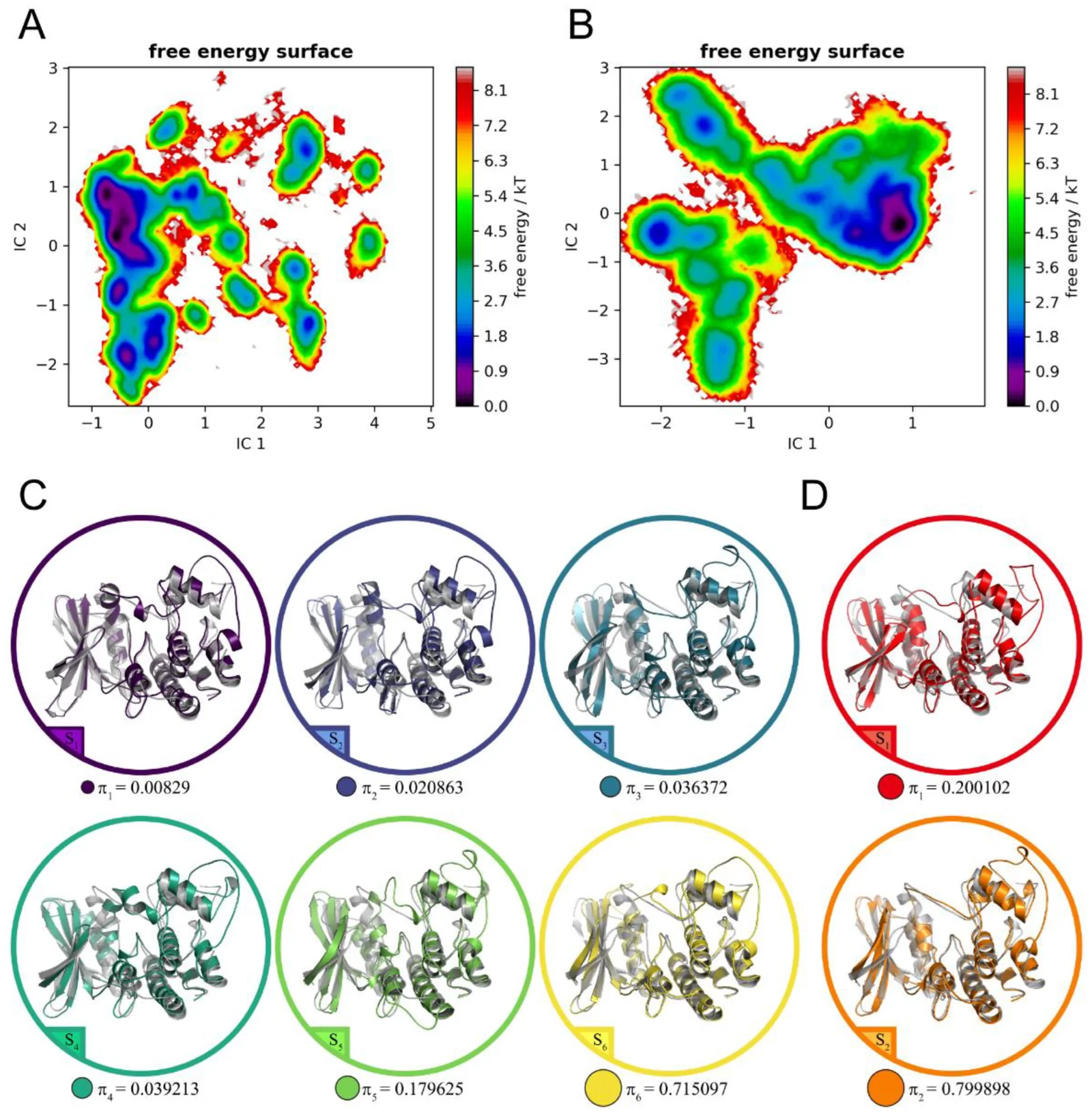
Markov State Model (MSM) analysis revealing metastable conformational states of CDK4 in bound and unbound conditions. (**A**) Two-dimensional free-energy landscape of the CDK4–**8i** complex constructed from TICA components IC1 and IC2; six metastable states (S1–S6) are identified, with lower free-energy basins (darker shading) representing more populated conformations. **8i** binding diversifies the accessible conformational space compared to the apo state. (**B**) Two-dimensional free-energy landscape of the apo CDK4 system showing two metastable states (S1–S2); the narrower energy landscape reflects more restricted conformational sampling in the absence of ligand. (**C**,**D**) Visualization of representative structures from each metastable state superimposed onto the CDK4 crystal structure (gray cartoon); each state is depicted by three representative snapshots (colored cartoons) to capture conformational diversity. Circle size reflects the equilibrium probability of each state, and region shading indicates the state-associated probability density. These panels highlight the progressive shift of the G-loop toward the N-terminus across the metastable states, which gradually exposes the cryptic allosteric pocket. Next, to investigate the structural changes in the G-loop region of the active site during the binding process, we performed a transition path analysis based on transition path theory (TPT) [22], using S1 as the initial state and S6 as the target state (Figure 7C; conformational structures for each path are provided in the Supplementary Materials). To further characterize the dynamic transitions between metastable states, the mean first passage time (MFPT) was computed from the constructed Markov state model (MSM). The MFPT represents the average time required to reach a target state from an initial state and has been widely applied in studies of protein folding kinetics and conformational transitions. As listed in Table 1, several transition paths from S1 to S6 were identified. The primary path, S1→S3→S6, accounts for 54.30% of the transitions, while the secondary path, S1→S3→S2→S6, contributes 27.15%. The remaining paths have relatively lower probabilities. The slower overall transition kinetics can be attributed to the first transition state S4, which exhibits a long MFPT of 365.3 ns, thereby influencing the entire conformational change process. Visualization of the metastable conformations along the main pathway (Figure 7A), together with the steady-state RMSD values computed for the path, reveals that during protein motion, the G-loop in the active site gradually shifts toward the N-terminal direction. As a result, the active pocket progressively enlarges.

To explore the conformational changes during protein movement, we then carried out large-scale aMD simulations of CDK4 before and after small molecule **8i** binding. Then we use PyEMMA to identify the key structural changes in the binding process [23]. Firstly, the backbone torsions were characterized to describe the overall topological structure of the protein, and using TICA to reduce the dimension of molecular simulation data. 100 steps were selected as the delay time and retained the first two components IC1 and IC2, which made the greatest contribution to the kinetic variance. We use IC1 and IC2 to construct two-dimensional energy landscape maps of combined and unintegrated systems respectively (Figure 6A,B). It shows that the protein will show more conformational changes when binding ligands. Using a k-means clustering algorithm for clustering, the whole simulation trajectory is clustered into 200 microstates. Then, the implicit time scale is used to calculate the lag time of the Markov model, and the implicit time scale curve converges when the lag time is about 40 ps. Using the PCCA algorithm, the micro-states are further clustered into six steady-state S1–S6 of the bound state and two steady-state S1 and S2 of the unbound state, and then the Chapman-Kolmogorov test verifies that the constructed MSM is Markov (Table 2). To identify the key structural features in the process of pocket change, we extracted representative conformations from each metastable state and superimposed them with CDK4 structures for further study (Figure 6C,D). To identify the structural change process of CDK4, we use protein root mean square deviation (RMSD is the coordinate deviation of a specific part of atoms relative to the reference structure, the smaller the difference, the smaller the RMSD) to evaluate and screen the initial and final states in MSM results. The RMSD values of each metastable conformation and CDK4 G-loop region were calculated, and the initial and final states of the structural changes in the bound state were determined to be S1 and S6, respectively. In the unbound state, the initial state was S1 (1.80 Å) and the final state was S2 (4.86 Å).

**Figure 7:**
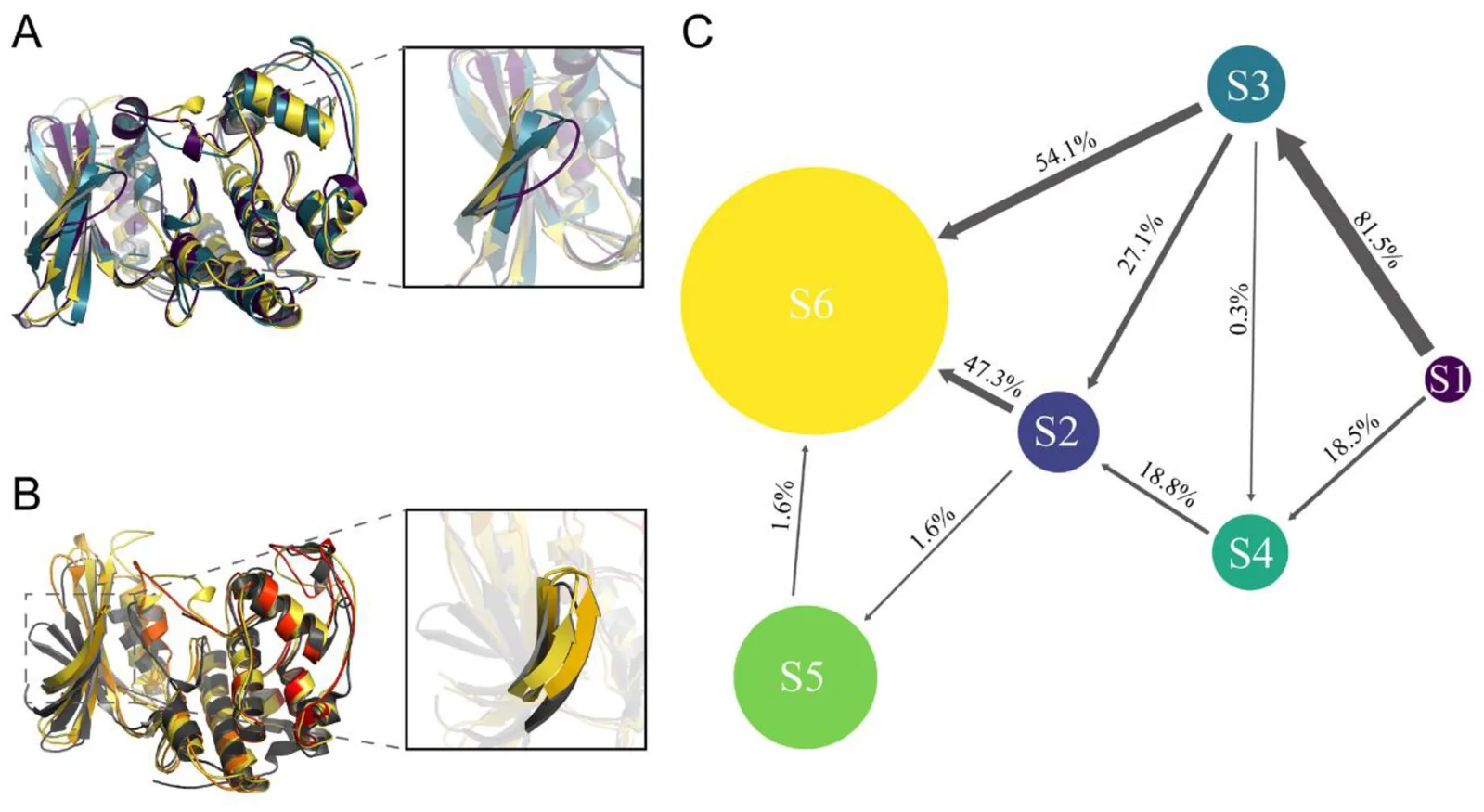
Conformational transition process of CDK4 in the ligand-bound state as revealed by MSM and transition path theory (TPT). (**A**) Superposition of representative structures along the dominant transition pathway (S1→S3→S6, accounting for 54.30% of total flux) with an inset illustrating the progressive G-loop displacement toward the N-terminus; this directional movement progressively enlarges the active-site pocket. (**B**) Structural alignment of the CDK4 final states (bound S6 and unbound S2) with the CDK2 inactive conformation (PDB ID: 5A14); G-loop RMSD values of 2.10 Å (bound) and 2.30 Å (unbound) relative to CDK2 demonstrate that CDK4 converges toward a CDK2-like inactive geometry regardless of ligand presence, though **8i** binding accelerates this transition (MFPT: 21.7 ns vs. 90.8 ns). (**C**) Flux diagram of reaction pathways between metastable states based on TPT; circle size represents the equilibrium probability of each state, and arrow thickness reflects the net reactive flux between states. The primary pathway (S1→S3→S6) and secondary pathway (S1→S3→S2→S6) together account for >81% of conformational transitions, with rate-limiting step at state S4 (MFPT = 365.3 ns).

By aligning the final state of bound and unbound states with the inactive conformation of CDK2, it is found that the conformational similarity of the superimposed conformation is very high. Then we also calculate the RMSD of the final state and CDK2 inactive state of the G-loop in bound and unbound states, which are 2.10Å and 2.30Å, respectively. Whether it binds to small molecules or not, after continuous movement, the G-loop of CDK4 tends to the shape of the inactive state of CDK2, which makes the active pocket similar (Figure 7B).

Comparing the MFPT of the two, the time from the initial state S1 to the final state S6 in the bound state is 21.7 ns, while the time from the initial state S1 to the final state S2 in the unbound state is 90.8 ns. The distance between N atom (**8i**) and H atom (Leu147) in the trajectory of the CDK4 binding state is calculated by using Amber’s CPPTRAJ packet. The contact distance between two atoms usually occurs at 250–300 ns. It is suggested that the interaction between small molecules and the residue Leu147 may accelerate the conformational change of the protein and shorten the timescale over which this change occurs.

## 3. Discussion

The crystal structure of CDK4 (PDB ID: 3G33) was selected as the initial model. We deliberately chose the apo form of this structure, as it represents the natural conformation of CDK4 before ligand binding, allowing us to study the conformational changes induced by ligands and the formation of potential hidden binding sites in the unbound state. Since the 3G33 structure does not contain the co-crystallized small molecule ligand, the complex conformation was obtained through the molecular docking method we reported previously [21]. The present study integrates accelerated molecular dynamics (aMD) simulations with Markov state model (MSM) analysis to investigate a previously unrecognized, putative allosteric mechanism in CDK4 that may be facilitated by the small molecule **8i**. Our findings, supported by complementary experimental validation, suggest that Leu147 may serve as an important regulatory residue in the proposed CDK4 allostery, and that **8i** binding is associated with a conformational transition in which the G-loop shifts toward the N-terminus, exposing a cryptic pocket with potential allosteric character and structural similarity to the inactive conformation of CDK2. These results carry significant implications for understanding CDK4 regulation and for the rational design of next-generation dual-target inhibitors.

### 3.1. Leu147 as a Potential Conformational Regulator of CDK4

Our computational and experimental results consistently suggest that Leu147 contributes to ligand binding and conformational stabilization of CDK4. The aMD simulations revealed that the wild-type CDK4–**8i** complex exhibited faster RMSD convergence and lower fluctuations compared to the L147A mutant complex, suggesting that **8i** binding accelerates conformational stabilization in a Leu147-dependent manner. RMSF analysis further demonstrated that ligand binding reduces residue flexibility in key structural regions—particularly around the G-loop—while the L147A mutation disrupts this stabilizing effect, leading to altered fluctuation patterns across multiple domains.

The MM/GBSA binding free energy calculations provided quantitative evidence that the Leu147Ala substitution substantially diminishes the binding affinity of **8i** for CDK4. This was corroborated by SPR experiments, which showed an approximately 2.7-fold increase in Kd (from 0.289 mmol/L to 0.774 mmol/L) upon mutation. Although these Kd values indicate moderate binding affinity overall—consistent with the micromolar-range IC50 values—the consistent trend across independent methods strongly supports the functional importance of Leu147. Notably, the DARTS assay provided direct biochemical evidence that **8i** confers proteolytic resistance to wild-type CDK4 but fails to protect the L147A mutant, confirming that Leu147 is essential for ligand-induced structural stabilization.

Structural analysis offered mechanistic insight into Leu147’s role. Although Leu147 does not form direct hydrogen bonds or π-stacking interactions with **8i**, its hydrophobic side chain provides steric support and maintains the geometric closure of the binding pocket. In the wild-type complex, **8i** is deeply embedded within a compact, well-defined pocket, with Leu147 contributing to a hydrophobic platform that constrains the ligand’s position. Upon mutation to alanine, the reduced side-chain volume creates excess cavity space, leading to ligand misalignment, partial solvent exposure, and disruption of the cooperative interaction network—including loss of key hydrogen bonds with neighboring residues. This finding is consistent with the concept of “hydrophobic platform” residues that indirectly stabilize ligand binding by maintaining the spatial organization of nearby polar residues, an analogy previously invoked for G protein-coupled receptors (GPCRs) and other regulatory kinases.

Based on the simulation results, we propose a working model in which Leu147 may contribute to ligand-associated conformational regulation. Upon **8i** entry into the binding pocket, minor steric repulsion with Leu147 may trigger side-chain displacement, which could propagate through a dynamic coupling network to reshape the binding site into a conformation more favorable for ligand accommodation. This proposed pre-organization mechanism could reduce the entropic penalty of binding and enhance affinity—a paradigm well-documented in allosterically regulated systems. The analogy to thermostabilized GPCRs, where key residues serve as structural anchors that prolong ligand residence time, further supports this interpretation.

### 3.2. G-Loop Dynamics and a Putative Cryptic Pocket with Potential Allosteric Character

The MSM analysis revealed a marked expansion of conformational space upon **8i** binding, with six metastable states (S1–S6) identified in the bound system compared to only two (S1–S2) in the apo system. This diversification indicates that **8i** does not merely stabilize a single conformation but rather facilitates sampling of multiple conformational substates, broadly analogous to the role ascribed to allosteric modulators in reshaping the free-energy landscape [24,25].

Transition path theory (TPT) analysis identified the dominant pathway S1→S3→S6 (54.30% of total flux) and the secondary pathway S1→S3→S2→S6 (27.15%), together accounting for over 81% of conformational transitions. Along this pathway, the G-loop undergoes a progressive shift toward the N-terminus, resulting in gradual enlargement of the active-site pocket. This directional movement is of particular significance because it represents a structural transition from the CDK4-typical active-site geometry toward a conformation resembling the inactive state of CDK2. The RMSD values of the G-loop region between the CDK4 final states and the CDK2 inactive conformation (PDB: 5A14) were 2.10 Å (bound) and 2.30 Å (unbound), indicating substantial structural convergence regardless of ligand presence.

The observation that CDK4 can adopt a CDK2-like inactive geometry has profound implications for the design of dual-target CDK2/CDK4 inhibitors. The pronounced spatial divergence between the active pockets of CDK2 and CDK4, which primarily originates from conformational differences in their respective G-loop domains, has long been recognized as a central challenge in developing such dual-target agents [14]. Our findings suggest that this divergence is not static but rather dynamic: the G-loop of CDK4 can transition to a conformation that narrows the structural gap between the two kinases, thereby creating a transient binding pocket capable of accommodating ligands designed for CDK2. This dynamic convergence provides a structural rationale for the moderate CDK2 inhibitory activity of **8i** (IC50 = 9409.8 nM) alongside its CDK4 activity (IC50 = 17,677.3 nM), as well as its negligible CDK6 inhibition—underscoring the selectivity profile unique to this compound.

### 3.3. ***8i*** as a Potential Modulator of CDK4 Conformational Dynamics

A particularly notable finding is the kinetic disparity between the bound and unbound systems. The mean first passage time (MFPT) from the initial to the final state was 21.7 ns in the bound system versus 90.8 ns in the unbound system, representing an approximately 4.2-fold acceleration of conformational transition upon **8i** binding. CPPTRAJ analysis further revealed that the contact distance between **8i** and Leu147 typically forms at 250–300 ns, temporally coinciding with the accelerated phase of conformational change. This suggests that the **8i**–Leu147 interaction may act as a kinetic catalyst that lowers the energy barrier for G-loop displacement, potentially channeling the conformational transition along the productive pathway.

Our simulations suggest that **8i** may influence the conformational landscape of CDK4 beyond simple orthosteric binding; however, additional studies are required to determine whether this represents a bona fide allosteric mechanism. By stabilizing intermediate states along the S1→S3→S6 pathway and promoting the G-loop shift, **8i** appears to facilitate the formation of a cryptic pocket that is not present in the ground-state structure. This mode of action is conceptually analogous to the cryptic allosteric site identified in SIRT6, where small-molecule binding induces the formation of a previously inaccessible pocket [25], and to the allosteric modulators of CDK4 described by Morris et al. [4], which regulate kinase activity through conformational mechanisms distinct from direct active-site blockade.

### 3.4. Implications for Overcoming CDK4/6 Inhibitor Resistance

CDK4/6 inhibitors, including palbociclib, ribociclib, and abemaciclib, have significantly improved therapeutic outcomes for hormone receptor-positive breast cancer. However, the development of acquired resistance remains a major limitation of this therapeutic strategy. Multiple resistance mechanisms have been reported, including activation of compensatory CDK2 signaling, Cyclin E amplification, loss or alteration of RB pathway regulation, and adaptive changes in cell-cycle control networks. Among these mechanisms, CDK2 activation has received considerable attention because it can bypass CDK4/6 inhibition and restore RB phosphorylation, allowing tumor cells to continue proliferating despite CDK4/6 blockade [11,12,13].

Current strategies to overcome resistance largely focus on combination therapies, particularly approaches targeting both CDK4/6 and CDK2. However, broad inhibition of multiple CDK family members may increase toxicity and limit therapeutic windows. Therefore, the development of selective CDK4 inhibitors represents an attractive alternative strategy. Selective suppression of CDK4 activity may preserve the antitumor effects associated with CDK4 pathway inhibition while potentially avoiding some adverse effects related to CDK6 inhibition, particularly hematological toxicity. Furthermore, selective CDK4 inhibitors may provide opportunities for rational combination with CDK2 inhibitors to simultaneously block complementary cell-cycle escape mechanisms [14].

Our study offers a potential solution to this challenge. The pocket exposed by the G-loop shift in CDK4 (a putative allosteric pocket) adopts a geometry similar to the CDK2 inactive conformation, suggesting that rationally designed ligands could target both pockets simultaneously or sequentially. The identification of Leu147 as a potential regulatory node further provides a specific target for structure-based drug design: compounds that engage Leu147 and promote the conformational transition could function as allosteric facilitators that sensitize CDK4 to co-administered CDK2 inhibitors, or as bifunctional agents that bridge both active sites. This concept of allosteric sensitization may represent a paradigm shift from traditional orthosteric inhibition strategies and may open new avenues for circumventing resistance. Although our structures reveal how ligands regulate CDK4 conformation, cellular studies are needed to link these changes to signaling, cell-cycle dynamics, and clinical outcomes in breast cancer.

### 3.5. Limitations and Future Perspectives

While our integrated computational and experimental approach provides supportive evidence for the proposed allosteric mechanism, several limitations should be acknowledged. First, the binding affinity of **8i** for CDK4 is moderate (k_d_ = 0.289 mmol/L, IC_50_ = 17,677.3 nM), which limits its direct therapeutic utility and suggests that structural optimization will be necessary to achieve pharmacologically relevant potency. Second, although DARTS and SPR experiments confirm the Leu147-dependent binding behavior, the mechanistic interpretations of signaling pathways and conformational propagation are derived primarily from molecular dynamics simulations, which inherently involve approximations in force-field parameters and sampling efficiency. An additional limitation of the present study is that the proposed ligand-associated conformational regulation was validated primarily through computational analyses and biochemical binding assays. Although mutagenesis, DARTS, and SPR experiments support the contribution of Leu147 to ligand binding and protein stabilization, the downstream biological consequences of these conformational changes have not yet been investigated in cellular systems. Future studies will therefore evaluate whether the proposed conformational regulation influences CDK4 kinase activity, RB phosphorylation, cell-cycle progression, and breast cancer cell proliferation. Such functional experiments will be essential for establishing the physiological relevance of the computational model. Future work should employ site-directed mutagenesis in cellular contexts, kinase activity assays with purified allosteric-state trapped conformations, and cryo-EM or X-ray crystallography to capture the cryptic pocket in the ligand-bound state. Additionally, structure-based optimization of **8i** derivatives, guided by the allosteric pocket geometry and the Leu147 interaction hotspot, may yield compounds with improved potency and selectivity profiles suitable for preclinical evaluation.

In summary, this study supports the proposed Leu147-mediated allosteric pathway in CDK4 that may be facilitated by the small molecule **8i**, potentially leading to G-loop displacement and cryptic pocket formation. The convergence of this pocket toward a CDK2-like geometry provides a structural foundation for dual-target inhibitor design and offers a potential strategy to overcome CDK4/6 inhibitor resistance in breast cancer. These findings provide a basis for allosteric modulator discovery that may be applicable to other kinases with structurally divergent active sites.

## 4. Materials and Methods

### 4.1. Molecular Docking

AutoDock 4 (version 4.2.6) was used for molecular docking. Firstly, the CDK4 (PDB ID: 3G33) structure was imported into SWISS-MODEL web server for residue repair [26]. This specific ligand-free (apo) structure was selected as the starting model because it represents the baseline conformational state of the kinase, and previous studies have successfully employed this structure in docking simulations to identify novel cryptic binding pockets and allosteric sites. The CDK4 and ligand structures were first converted to PDBQT format using AutoDockTools (version 1.5.7) and then loaded into AutoGrid 4 (version 4.2.6).

### 4.2. Classical Molecular Dynamics (MD) Simulation

Classical MD simulations predict the physical movements of atoms and molecules over time by numerically solving Newton’s equations of motion. Empirical force fields are used to calculate the potential energy of the system, allowing us to evaluate the dynamic stability, conformational changes, and binding mechanisms of the protein-ligand complexes under simulated physiological conditions.

#### 4.2.1. GROMACS

GROMACS 2021.4 [27] was used to simulate MD. The Amber99SB-ILDN force field was used to prepare protein topology and parameter files, and the GAFF2 force field was used to describe small molecules. These force fields define the mathematical parameters for atomic interactions (such as bond stretching, angle bending, and non-bonded interactions), which are crucial for accurately depicting the dynamic behavior of the system. The RESP2 (Restrained Electrostatic Potential) charge of small molecules was calculated by Multiwfn 3.8 combined with ORCA [28], providing a highly accurate electrostatic representation of the ligand necessary for evaluating binding affinities. The protein-ligand complex was placed in a dodecahedral box and solvated using the TIP3P water model and SPC216 water molecules to mimic the physiological aqueous environment. The distance between the protein edge and the box was at least 10 Å. Enough Na+ and Cl- ions were added to neutralize the system to prevent artifactual electrostatic interactions across periodic boundaries. The steepest descent algorithm eliminated torsional strain and spatial conflicts by setting Fmax to 1000.0 kJ·mol^−1^·nm^−1^ to minimize the energy of the system, ensuring a stable starting structure without unnatural atomic overlaps that could crash the simulation. Then the NVT (constant Number, Volume, Temperature) and NPT (constant Number, Pressure, Temperature) equilibrations of 100 ps were performed respectively. These phases allow the solvent and ions to relax around the complex. The heavy atoms of the protein skeleton and ligand were restricted by using the Berendsen thermostat (V-rescale) and the Berendsen barostat. The balance of NVT and NPT gradually heated the system to 300 K and reached the pressure of 1 bar, establishing realistic thermodynamic conditions. After the position constraint was removed, the leap-frog integrator was used to run the MD of 200 ns. The particle mesh Ewald (PME) scheme was used to estimate the long-range electrostatic interaction, which accurately calculates Coulombic forces across the periodic box. The cutoff distance of van der Waals force and Coulomb interaction was set to 12.0 Å, balancing computational efficiency and physical accuracy. The hydrogen bonds were constrained by the LINCS algorithm, which removes high-frequency bond vibrations, allowing the minimum time step to be set to 2.0 fs to accelerate the simulation without losing thermodynamic stability.

#### 4.2.2. Assisted Model Building with Energy Refinement (Amber)

Amber(version 2024) [28] was used to simulate MD. The Amber99SB-ILDN force field was used to parameterize CDK4 and the GAFF2 force field was used to describe small molecules. The RESP2 charge of small molecules was calculated by Multiwfn 3.8 combined with ORCA [29]. The protein-ligand complex was placed in a dodecahedral box and solvated using the TIP3P water model. The distance between the protein edge and the box was at least 10 Å. Counter ions were added to neutralize the protein.

Two rounds of energy minimization were carried out after preliminary preparation to remove steric clashes. First, the energy of water and ions was minimized by 5000 steps (the steepest descent in 2000 steps, and then the conjugate gradient minimization in 3000 steps), and the protein skeletons and ligands were constrained by 500 kcal/(mol·Å^2^) position constraints. Then, without any restriction, the 4000 steepest descent and 6000 conjugate gradient minimization cycles were carried out for the whole composite system. After that, the simulation system was heated to 300 K in 500 ps, then the 700 ps was balanced under the condition of NVT, and the 10 kcal/(mol·Å^2^) constraint was imposed on the protein and ligand structure, then the global equilibrium of 5 ns was carried out. Finally, the cMD (classical MD) mode of 50 ns was run at the time step of 2 fs at 1 atmospheric pressure and 300 K. After the completion of the simulation, the potential energy and the dihedral energy were calculated to prepare for further accelerated MD (aMD) simulation, which adds a boost potential to reduce energy barriers and enhance conformational sampling. To adjust the temperature of the system, the Langevin dynamics with 1 ps^−1^ collision frequency was applied, and the Berendsen thermostat with isotropic position scaling was used to adjust the pressure of the system. In the cMD process, the long-range electrostatic interaction was modeled by the particle mesh Ewald (PME) method, and the cutoff values of short-range electrostatic interaction and van der Waals forces were set to 10 Å. In addition, the SHAKE algorithm was used to constrain the covalent bonds involving hydrogen atoms, permitting a larger integration time step without system instability.

### 4.3. Molecular Mechanics Poisson–Boltzmann Surface Area (MMGBSA) Binding Free Energy

Gmx_MMGBSA(2021.6) package was used to calculate the free energy of the single trajectory and Amber99SB-ILDN force field based on GROMACS [30,31]. The tool allows the calculation of free energy using MM/PBSA or GBSA (molecular mechanics/Poisson-Boltzmann or generalized born surface area) methods. The free energy of MM/PB (GB) SA binding between protein and ligand in the solvent can be expressed as follows:

(1) $$∆G_{bind}=∆G_{complex}-\left(∆G_{protein}+∆G_{ligand}\right)$$

The free energies of proteins, ligands, or complex components can be calculated as follows:

(2) $$∆G_{bind}=∆E_{MM}+∆G_{solv}-T∆S$$

(3) $$∆E_{MM}=∆E_{bond}+∆E_{vdW}+∆E_{elec}$$

(4) $$∆G_{solv}=∆G_{PB/GB}+∆G_{SA}$$

In the above Equations, ∆*E_MM_*, ∆*G_solv_* and *T*∆*S* are the changes of molecular mechanical energy, solvation free energy and conformational entropy of gas phase when ligands are combined. ∆*E_bond_* is the energy of bonding interactions, which was calculated to be zero in the dynamic simulation, and ∆*E_vdw_* and ∆*E_elec_* are the van der Waals and electrostatic interaction energies, respectively. The ∆*G_solv_* is calculated from the polar ∆*G_(PB⁄GB)_* (electrostatic solvation energy) and non-polar ∆*G_SA_* between the solute and the continuous solvent. The polar contribution was estimated by GB-OBC1 model, while non-polar energy was estimated by solvent accessible surface area (SASA). In this work, snapshots sampled from the MD trajectories of each protein-ligand complex were used to calculate the binding free energy using the MM/GBSA method because it is computationally efficient and requires fewer resources. In the case of comparing the binding state of ligands to the same protein, the entropy (∆*S*) effect has the least effect on the total energy, so it was ignored.

### 4.4. Accelerated Molecular Dynamics (aMD)

aMD was performed to enhance sampling and cover a larger conformational space after 50 ns cMD simulation. Throughout the aMD process, “lifting energy” was added to the simulation system to reduce energy barriers and achieved easier conversion between various conformations in the free energy landscape.

In Equations (5) and (6), the potential energy of the system is calculated as *V_r_*, and the corresponding threshold is determined by the size of the system and its normal energy value as *E_thresh_*. In the aMD process, when the instantaneous potential energy is less than *E_thresh_*, it is increased by ∆*V_r_* to reach V_r_^*^. Otherwise, the original value *V_r_* is used.

(5) $$V_{r}^{*}=V_{r};\;V_{r}\geq E_{thresh}$$

(6) $$V_{r}^{*}=V_{r}+∆V_{r};\;V_{r}<E_{thresh}$$

where ∆*V_r_* is defined in Equation (7) and depends on *E_thresh_* and the acceleration parameter α.

(7) $$∆V_{r}=\frac{{\left(E_{thresh}-V_{r}\right)}^{2}}{E_{thresh}-V_{r}+\alpha }$$

In the aMD process based on Equations (8)–(11), the “double boost” technique was used to increase the potential energy and dihedral energy [24]. In Equations (8) and (10), *E_threshP_* represents the potential energy threshold, while *E_threshD_* represents the dihedral energy threshold; in Equations (9) and (11), *α_P_* and *α_D_* represent the acceleration parameters of potential energy and dihedral energy, respectively. The average total energy (*E_tot avg_*) and the average dihedral energy (*E_dih avg_*) were calculated in cMD simulation and then used in aMD. *N_atoms_* and *N_residues_* represent the number of atoms and residues in the system.

(8) $$E_{threshP}=E_{tot\;avg}+N_{atoms}\times 0.16\;kcal/mol$$

(9) $$\alpha_{P}=N_{atoms}\times 0.16\;kcal/mol$$

(10) $$E_{threshD}=E_{dih\;avg}+N_{residues}\times 4\;kcal/mol$$

(11) $$\alpha_{D}=N_{residues}\times 4/5\;kcal/mol$$

The final structure was extracted from the cMD simulation trajectory as the starting structure of the aMD simulation, and then aMD was run 500 ns at a random initial speed. To strengthen the sampling, five independent repeated simulations of the combined and uncombined systems were carried out, and the cumulative simulation trajectories of 2.5 μs were obtained. The energy calculation and system environment parameter setting of aMD were consistent with those used in cMD. The simulation trajectory analysis was performed using Amber’s CPPTRAJ package [25].

### 4.5. Construction and Verification of MSM

In order to construct the MSM, the backbone torsions of the protein were used as the characteristic input of PyEMMA [32]. Time-lagged independent component analysis (TICA) was used to transform the initial features [33]. Next, the k-means method was used to cluster the whole trajectory. Then the MSM was constructed by selecting the appropriate delay time based on the implicit time scale test. Perron-cluster cluster analysis (PCCA) model was used to cluster it into multiple metastable states, and the Markov property was verified by Chapman-Kolmogorov test. For each metastable state, the corresponding representative structure was extracted from the simulation trajectory according to the similarity score *S_ij_* calculated by Equation (12).

(12) $$S_{ij}=e^{-dij/dscale}$$

Here, *d_ij_* reflects the RMSD comparing snapshot structures *i* and *j*, and *d_scale_* is the standard deviation of d. By iterating each frame in pairs in the whole trajectory, the highest estimated structure of *S_ij_* was regarded as a representative conformation, and clustering extraction was carried out.

The hidden time scale test was carried out to test the Markov characteristics of our simulation system and the MSM constructed from it. Firstly, the number of k-means centers and the maximum number of iterations were defined, and then the corresponding multiple transition probability matrix (TPM) with different lag times were constructed. The TPM represents the transition probability between all microstates, and it also determines the relaxation time scale or implicit time scale according to Equation (13).

(13) $$\tau_{i}=-\tau /ln\lambda_{i}$$

where *τ* denotes the lag time of TPM and *λ_i_* represents the *i*th eigenvalue corresponding to TPM with lag time *τ*. When the microstate transition is relaxed for the *i*th time, the corresponding implied timescale *τ_i_* can be calculated from Equation (13).

The implied timescale is derived from the feature decomposition of TPM, and the orders of eigenvalues reflects the sequence of transformation. Therefore, the first eigenvalue *λ_1_* corresponds to the slowest transformation, and so on. With the increase of *τ***,** different *τ_i_* were calculated for Markov attribute verification. When *τ_i_* begins to stabilize with the increase of *τ*, that is, the transition between microscopic states becomes independent of the lag time, which indicates that the corresponding MSM is Markovian [34].

### 4.6. Drug Affinity Responsive Target Stability

Drug affinity responsive target stability (DARTS) is a reliable technique for identifying novel protein targets of small molecules [35]. The principle of DARTS is based on the increased proteolytic resistance of a target protein upon ligand binding, which stabilizes the protein structure. A 10× TNC buffer was prepared by mixing 500 μL of stock TNC solution with 100 μL of 1 M Tris-HCl (pH 8.0), 100 μL of 5 M NaCl, 100 μL of 1 M CaCl_2_, and 300 μL of ultrapure water. Recombinant human CDK4 and CDK4^L147A^ were purchased from AtaGenix Biotech (AtaGenix, Wuhan, China).

For the DARTS assay, 1 μL of 1 mM **8i** was added to 99 μL of protein solution, while 1 μL of DMSO was added to the control group. The mixtures were gently shaken and incubated at room temperature for 20 min. Subsequently, a streptomycin protease working solution (1:1000 dilution) was prepared in TNC buffer. Each sample was aliquoted into 20 μL portions, followed by the addition of 2 μL of the protease working solution. For the undigested control, 2 μL of 1× TNC buffer was substituted for the protease.

The samples were incubated at room temperature for 15 min to allow proteolysis. After digestion, 5.5 μL of 5× loading buffer was added, and the samples were heated at 100 °C for 10 min. The protein stability was subsequently analyzed by Western blotting.

### 4.7. Western Blot

A 10% sodium dodecyl sulfate–polyacrylamide gel electrophoresis (SDS–PAGE) gel was prepared and assembled into the electrophoresis apparatus, and protein samples were loaded at 200 ng per lane. Once the bromophenol blue dye front migrated to the lower edge of the gel, the proteins were transferred onto a polyvinylidene fluoride (PVDF) membrane using a standard wet-transfer procedure. The membrane was subsequently blocked in 5% skimmed milk or 5% bovine serum albumin (BSA) for 1.5 h at room temperature, followed by incubation with the appropriately diluted primary antibody overnight at 4 °C. The next day, the membrane was washed thoroughly and incubated with the corresponding horseradish peroxidase (HRP)-conjugated secondary antibody for 2 h at room temperature. After a final washing step, an enhanced chemiluminescence (ECL) substrate was evenly applied to the membrane surface, and the protein bands were visualized using a chemiluminescence imaging system. All results were recorded for analysis. The quantitative data are presented as the mean ± standard deviation (SD). DARTS-Western blot was performed in independent replicate experiments to confirm reproducibility.

### 4.8. Detection of CDKs Activity In Vitro

The ADP-Glo assay was employed to determine the inhibitory activities of the compound **8i** against CDK2, CDK4, CDK6. A reaction buffer consisting of 50 mM HEPES (pH 7.5), 10 mM MgCl_2_, 0.1 mg/mL BSA, 2 mM DTT, and 1% DMSO was used for all dilutions. Recombinant CDK and ATP were diluted to 2.2 μg/mL and 250 μM, respectively, in this buffer. Serial dilutions of the test compound were prepared across five concentrations (6 × 10^−2^ to 6 × 10^−10^ M). For the assay, 1 μL of **8i**, 2 μL of ATP, and 2 μL of enzyme were combined in a 96-well plate to start the reaction. After 1 h at 37 °C, 5 μL of ADP-Glo reagent was added, followed by a 40-min room-temperature incubation. The addition of 10 μL kinase detection reagent and a final 45-min incubation preceded luminescence reading on a multi-function microplate reader.

### 4.9. The Procedure for the Synthesis of Target Compound ***8i***

**Scheme 1:**
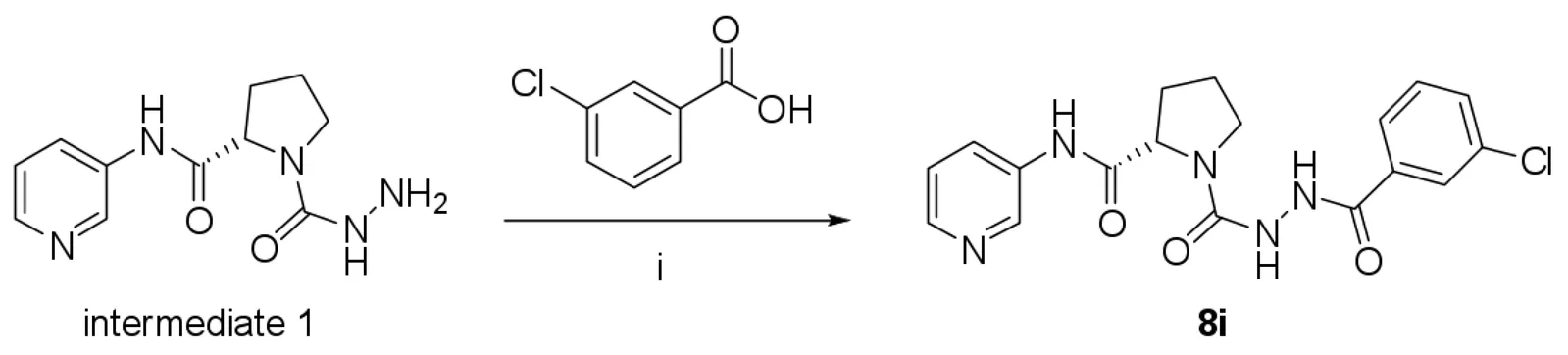
Reagents and conditions: (i) CH_3_SO_2_Cl, Et_3_N, r.t., 6 h.

A solution of 3-chlorobenzoic acid (1.93 mmol) and triethylamine (4.01 mmol) in acetonitrile (15 mL) was treated with methanesulfonyl chloride (2.00 mmol). All reagents were purchased from Aladdin Biochemical Technology Co., Ltd. (Shanghai, China). The reaction mixture was stirred at −5 °C in an ice bath for 1.5 h. Subsequently, this solution was added dropwise to a mixture of 1-(hydrazinecarbonyl)-*N*-(pyridin-3-yl)pyrrolidine- 2-carboxamide (intermediate 1, 1.93 mmol) in acetonitrile (10 mL). The resulting mixture was stirred at room temperature for 6 h, and the reaction progress was monitored by TLC. Upon completion, the solvent was removed under reduced pressure. The residue was partitioned between water and ethyl acetate. The organic layer was separated, dried over anhydrous Na_2_SO_4_ (Sinopharm Chemical Reagent Co., Ltd., Shanghai, China), and concentrated in vacuo. The crude product was purified by silica gel column chromatography to afford the target compound **8i** in pure form (Scheme 1).

High-performance liquid chromatography (HPLC) analysis was performed using an Agilent 1260 (Agilent Technologies, Santa Clara, CA, USA) Infinity II system equipped with a variable wavelength detector (VWD). Separation was achieved on an Agilent Poroshell HPH-C18 column (4.6 × 150 mm, 2.7 µm; Agilent Technologies, Santa Clara, CA, USA) maintained at 25 °C. The mobile phase consisted of water containing 0.1% trifluoroacetic acid (TFA) (solvent A) and acetonitrile containing 0.1% TFA (solvent B), delivered at a flow rate of 1.0 mL/min. The injection volume was set to 10 µL. Gradient elution was employed as follows: 0–100% solvent B over 80 min. Detection was carried out at a wavelength of 288 nm. Data acquisition and processing were performed using Agilent OpenLAB CDS ChemStation Edition (Agilent Technologies).

A light yellow solid, yield: 24.81%, M.p. 211.3–216.4 °C, [α]^20^_D_ = 138.19 (C = 0.05, CH_3_OH). ^1^H-NMR (500 MHz, DMSO-*d_6_*) *δ* 10.65 (s, 1H), 10.21 (s, 1H), 8.71 (s, 1H), 8.41 (d, *J* = 5.4 Hz, 1H), 8.17 (d, *J* = 4.3 Hz, 1H), 7.99–7.89 (m, 2H), 7.85 (d, *J* = 7.7 Hz, 1H), 7.64 (d, *J* = 7.9 Hz, 1H), 7.55 (t, *J* = 6.8 Hz, 1H), 7.25 (dd, *J* = 8.0, 4.7 Hz, 1H), 4.44 (d, *J* = 8.7 Hz, 1H), 3.67 (s, 1H), 3.49 (d, *J* = 7.7 Hz, 1H), 2.20–2.15 (m, 1H), 2.06 (d, *J* = 17.8 Hz, 3H). ^13^C-NMR (125 MHz, DMSO-*d_6_*) *δ*171.71, 163.68, 160.97, 153.36, 142.11, 141.10, 136.96, 134.47, 134.35, 130.58, 129.99, 126.36, 126.03, 122.96, 121.48, 117.13, 58.62, 46.31, 29.77, 23.97.

### 4.10. Statistical Analysis

Data are expressed as mean ± SEM. Comparisons were made using one-way ANOVA or two-tailed unpaired Student’s *t*-test (GraphPad Prism 10). *p* < 0.05 was considered significant. All Western blotting assays were repeated with at least three independent biological replicates, and band intensities were quantified via densitometry.

## 5. Conclusions

Based on the unique binding mode between the small molecule **8i** and CDK4, we employed aMD simulations together with MSM analysis to uncover a computationally-derived allosteric pathway in CDK4, in which Leu147 emerged as a potential regulatory residue. Supporting biochemical experiments demonstrated that mutation of Leu147 markedly reduced ligand binding and ligand-induced structural stabilization, providing experimental support for the computational model. Notably, the proposed allosteric pathway may give rise to a novel small-molecule binding pocket capable of accommodating larger ligands. This dual-targeting potential offers a promising strategy for over-coming resistance to CDK4 inhibitors. While our computational models and preliminary binding assays strongly suggest this allosteric regulation, we acknowledge the inherent limitations of interpreting signaling mechanisms primarily from molecular dynamics simulations and structural modeling. Future functional cellular assays will be necessary to fully validate the biological consequences of this proposed mechanism. Overall, this study presents a new framework for designing allosteric modulators and may facilitate the development of next-generation CDK4 inhibitors for breast cancer therapy.

## Figures and Tables

**Table 1:** Enzymatic inhibitory activities of novel compounds.

Compound	IC_50_ (nM) ^1^			Selectivity
	CDK6	CDK4	CDK2	CDK4/CDK2
**8i**	--	17,677.3 ± 2623.8	9409.8 ± 1874.3	1.9

^1^ The IC_50_ values are an average of two independent test.

**Table 2:** Transition pathways and their relative flux proportions for CDK4 bound-state conformational changes identified by transition path theory (TPT). Each pathway represents a sequence of metastable states (S1–S6) from the initial state (S1) to the final state (S6), with the associated mean first passage time (MFPT, ns) and percentage of total reactive flux. The dominant pathway S1→S3→S6 accounts for 54.30% of transitions, while S1→S3→S2→S6 contributes 27.15%.

	Path	Percentage
Path1	S1-S3-S6	54.30%
Path2	S1-S3-S2-S6	27.15%
Path3	S1-S4-S2-S6	17.30%
Path4	S1-S4-S2-S5-S6	1.25%

## Data Availability

The original contributions presented in this study are included in the article/Supplementary Material. Further inquiries can be directed to the corresponding author(s).

## Supplementary Materials

The following supporting information can be downloaded at: https://www.mdpi.com/article/10.3390/ph19081166/s1, Figure S1: Graphic abstract. Figure S2: Pre Molecular Dynamics RMSD Data. Figure S3: Molecular Dynamics RMSD Data. Figure S4: Molecular Dynamics RMSF Data. Figure S5: Allosteric relationship of CDK4 protein. Figure S6: WB raw data of CDK4. Figure S7: WB raw data of β-actin. Figure S8: ^1^H-NMR spectrum of compound **8i**. Figure S9: ^13^C-NMR spectrum of compound **8i**. Figure S10: Ramachandran plot of the energy-minimized CDK4 mutant model. Figure S11: Chiral Isomer RMSD Comparison Plot. Figure S12: Design strategy of compound **8i**. Figure S13: Chromatogram of **8i**. Table S1: Binding energy of wild-type and mutant protein-ligand complexes. Table S2: The water box information in the molecular dynamics research. Table S3: Enzymatic inhibitory activities of the compounds in our previous studies. Table S4: Chromatographic Parameters.
