# Supplementary material for: Proposed Allosteric Inhibition of Cyclin-Dependent Kinase 4 by a Proline-Derived Acylsemicarbazide Compound with Antiproliferative Activity in Breast Cancer Cells
Source: Pharmaceuticals (Basel). 2026 Jul 26;19(8):1166. doi: 10.3390/ph19081166 (PMC13516810; doi:10.3390/ph19081166)
Supplement: Supplementary file 1 [file pharmaceuticals-19-01166-s001.zip › pharmaceuticals-4370690-supplementary.pdf]

Supplementary Material

Supplementary Figures and Tables

Figure S1: Graphic abstract

Figure S2: Pre Molecular Dynamics RMSD Data

Figure S3: Molecular Dynamics RMSD Data

Figure S4: Molecular Dynamics RMSF Data

Figure S5: Allosteric relationship of CDK4 protein

Figure S6: WB raw data of CDK4

Figure S7: WB raw data of β-actin

Figure S8: ^{1}H-NMR spectrum of compound 8i.

Figure S9: ^{13}C-NMR spectrum of compound 8i.

Figure S10: Ramachandran plot of the energy-minimized CDK4 mutant model.

Figure S11: Chiral Isomer RMSD Comparison Plot

Figure S12: Design strategy of compound 8i.

Figure S13. Chromatogram of 8i

Table S1: Binding energy of wild-type and mutant protein-ligand complexes

Table S2: The water box information in the molecular dynamics research

Table S3. Enzymatic inhibitory activities of the compounds in our previous studies

Table S4. Chromatographic Parameters

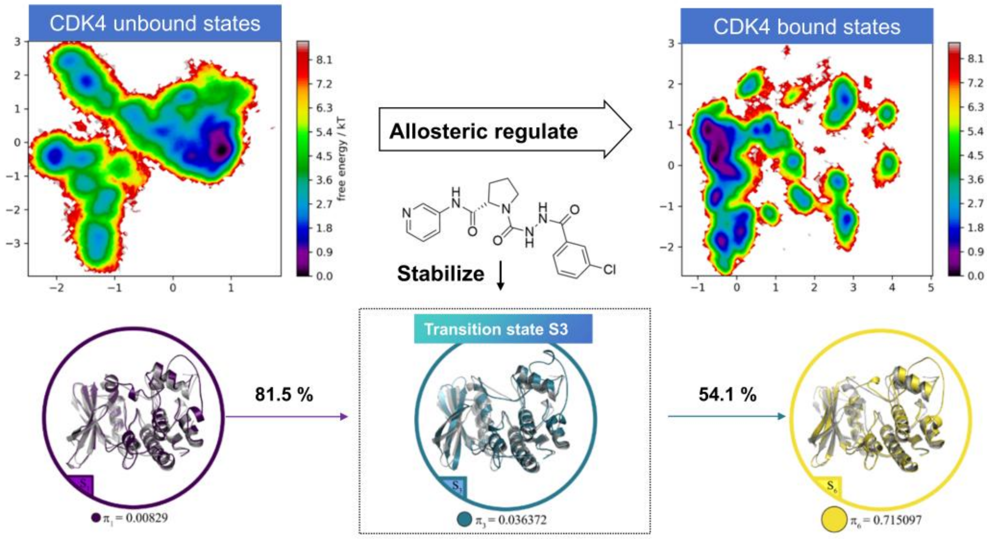

Figure S1: Graphic abstract

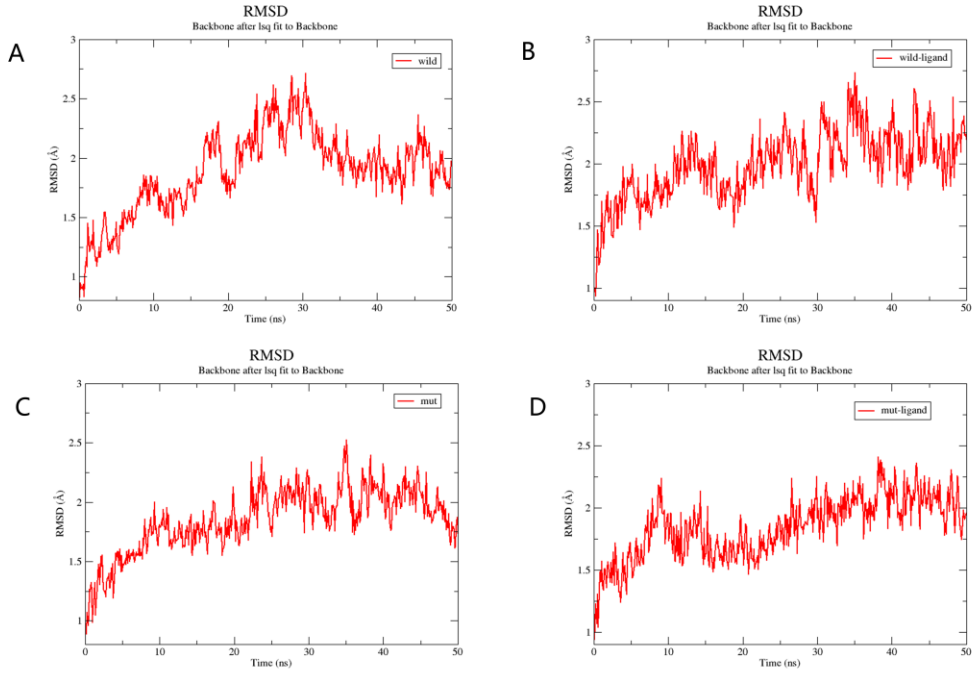

Figure S2. Pre Molecular Dynamics RMSD 50 ns Data. A: Time evolution of Pre RMSD for the wild-type protein. B: Time evolution of Pre RMSD for wild-type protein–ligand complex. C: Time evolution of Pre RMSD for the mut type protein. D: Time evolution of Pre RMSD for the mut-type protein–ligand complex.

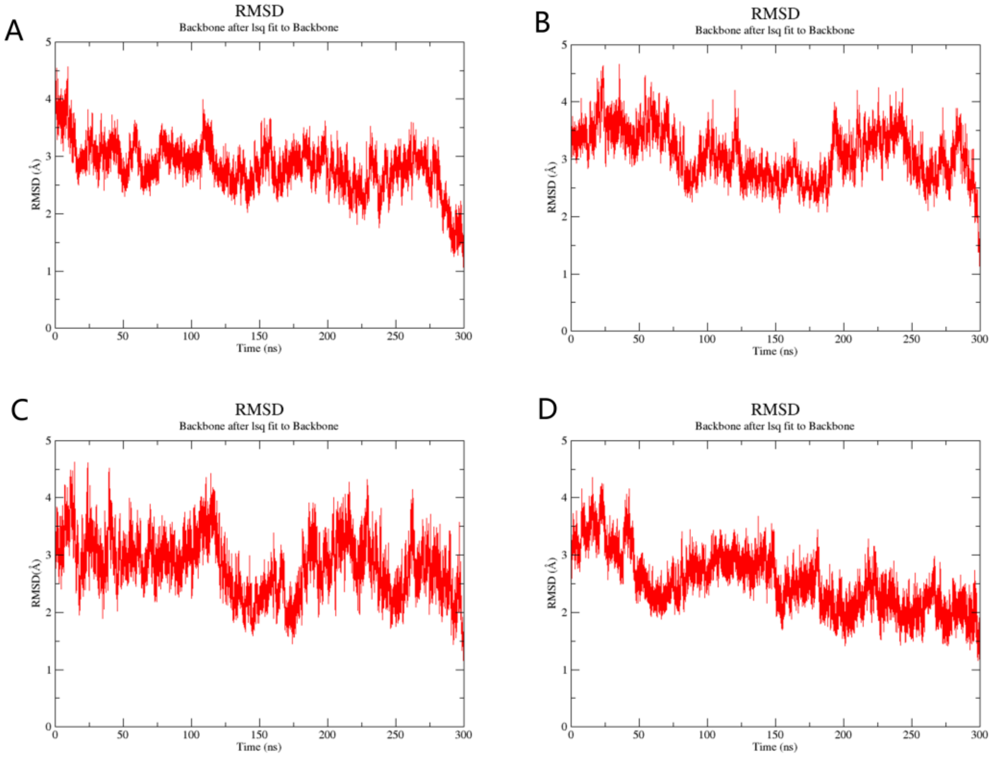

Figure S3. Molecular Dynamics RMSD Data. A:Time evolution of RMSD for the wild-type protein.B: Time evolution of RMSD for the mut type protein.C: Time evolution of RMSD for the wild-type protein–ligand complex.D: Time evolution of RMSD for the mut-type protein–ligand complex.

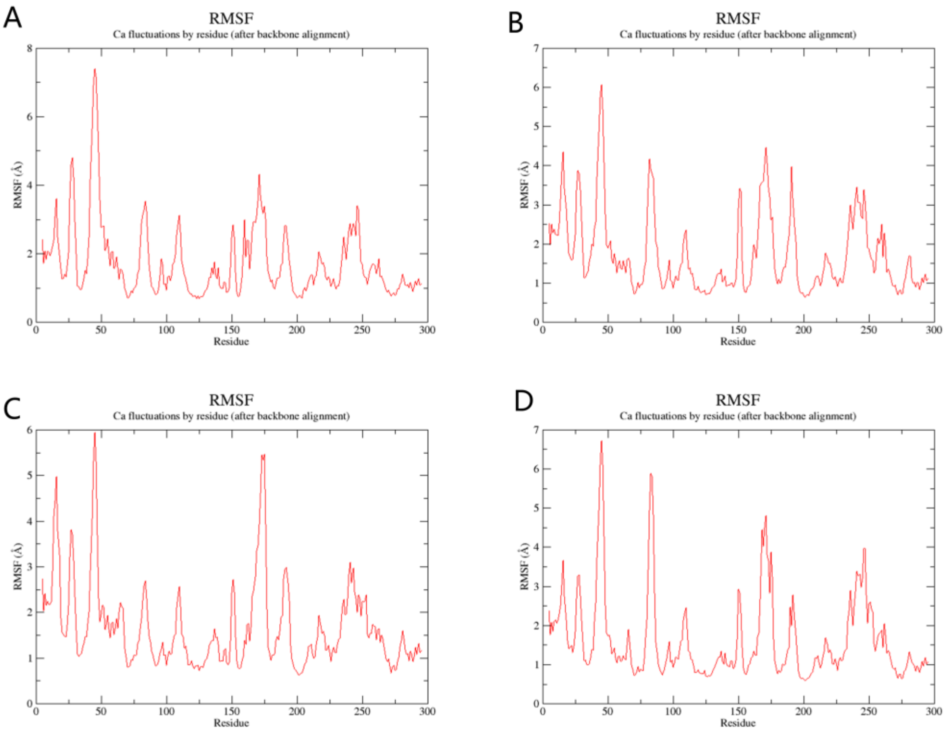

Figure S4. Molecular Dynamics RMSF Data. A: RMSF of amino acid residues in the wild-type protein a. B: RMSF of amino acid residues in the mut-type protein. C: RMSF of amino acid residues in the wild-type protein–ligand complex. D: RMSF of amino acid residues in the mut-type protein–ligand complex.

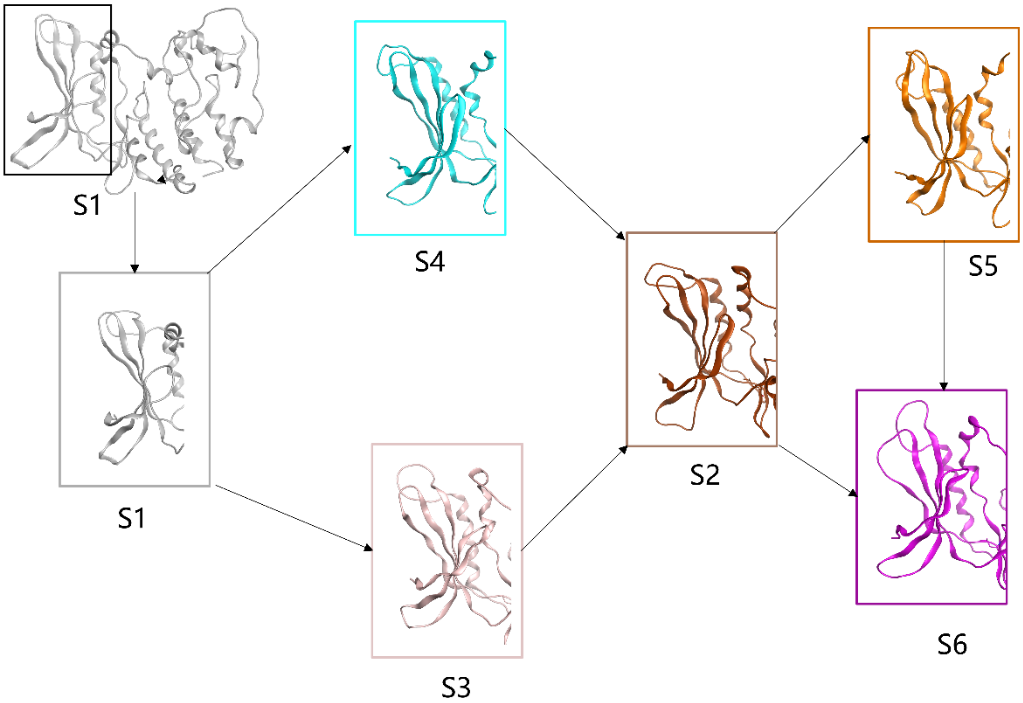

Figure S5. Allosteric relationship of CDK4 protein

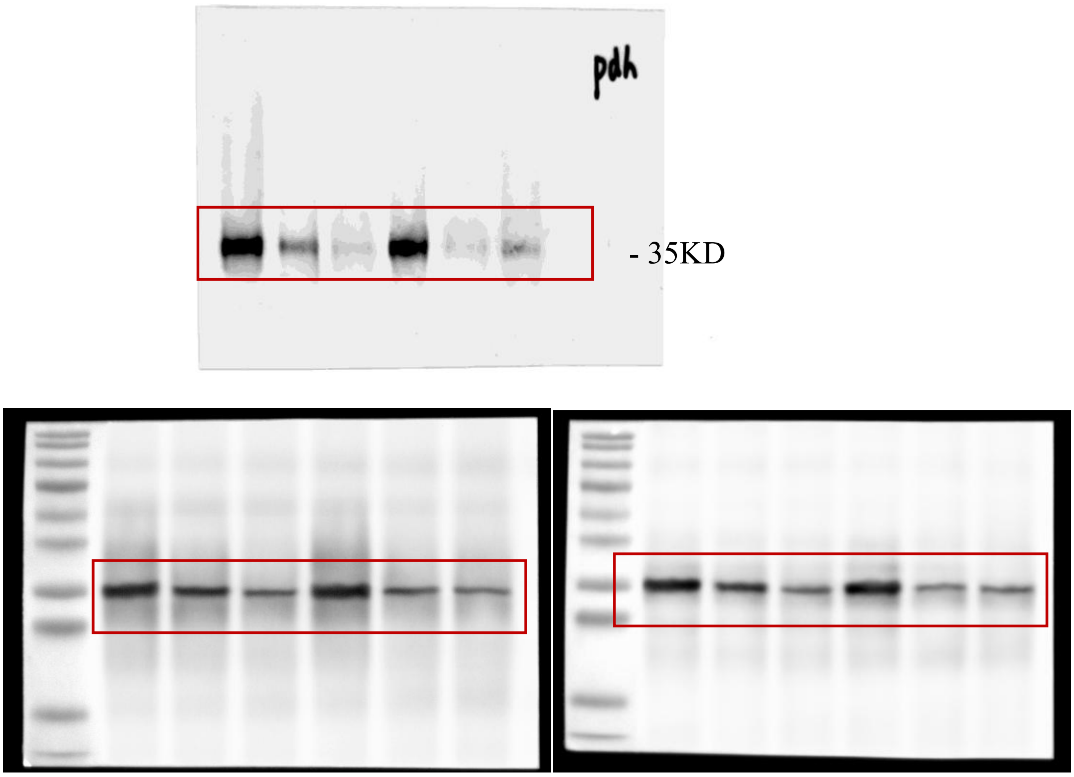

Figure S6. WB raw data of CDK4

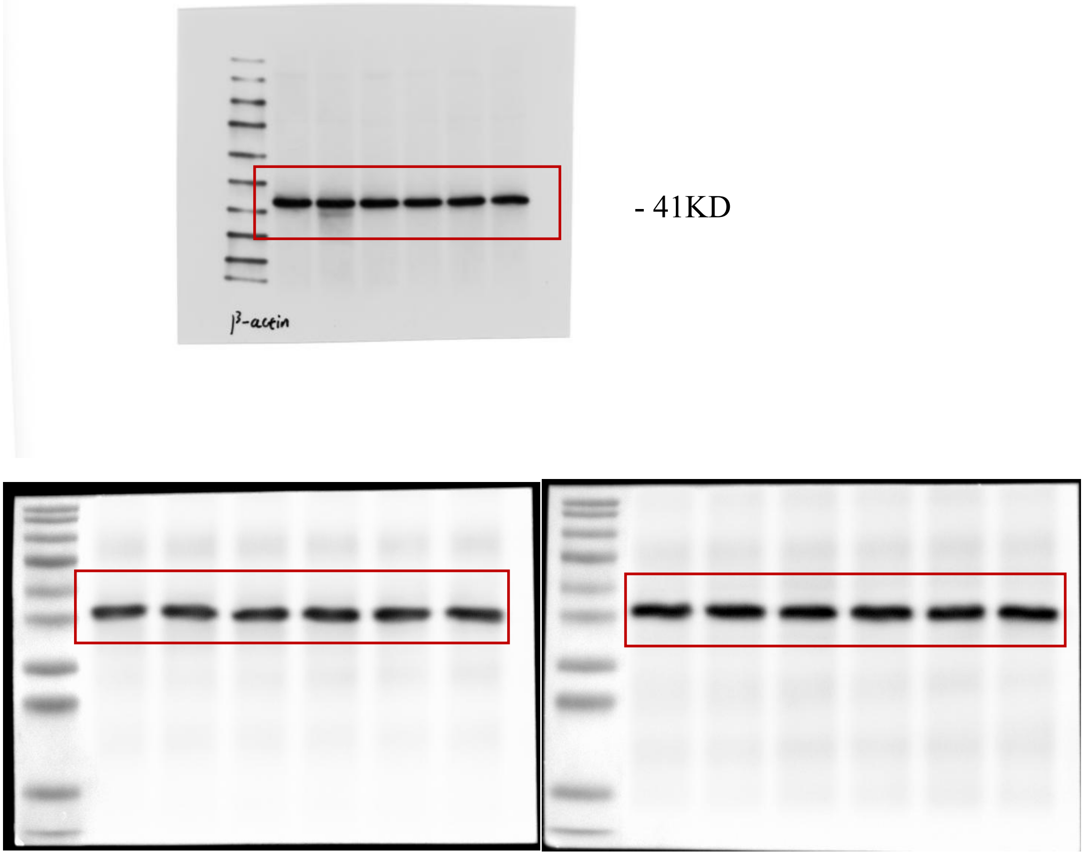

Figure S7. WB raw data of β-actin

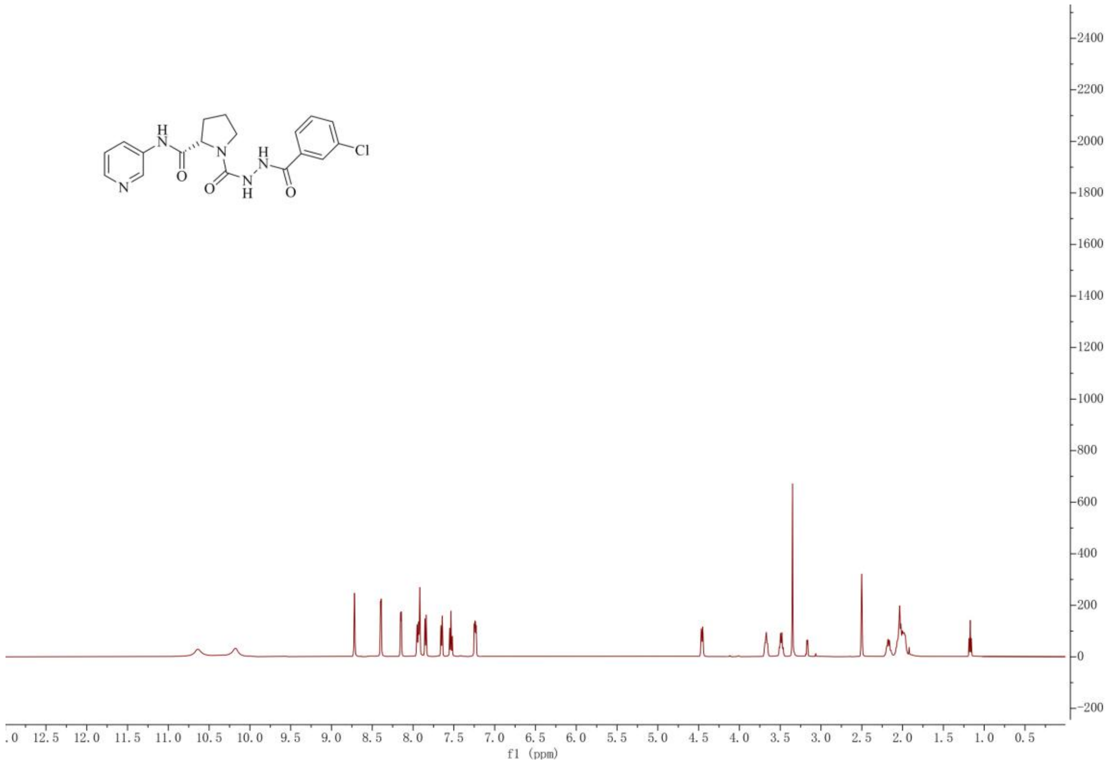

Figure S8. ^{1}H-NMR spectrum of compound **8i**.

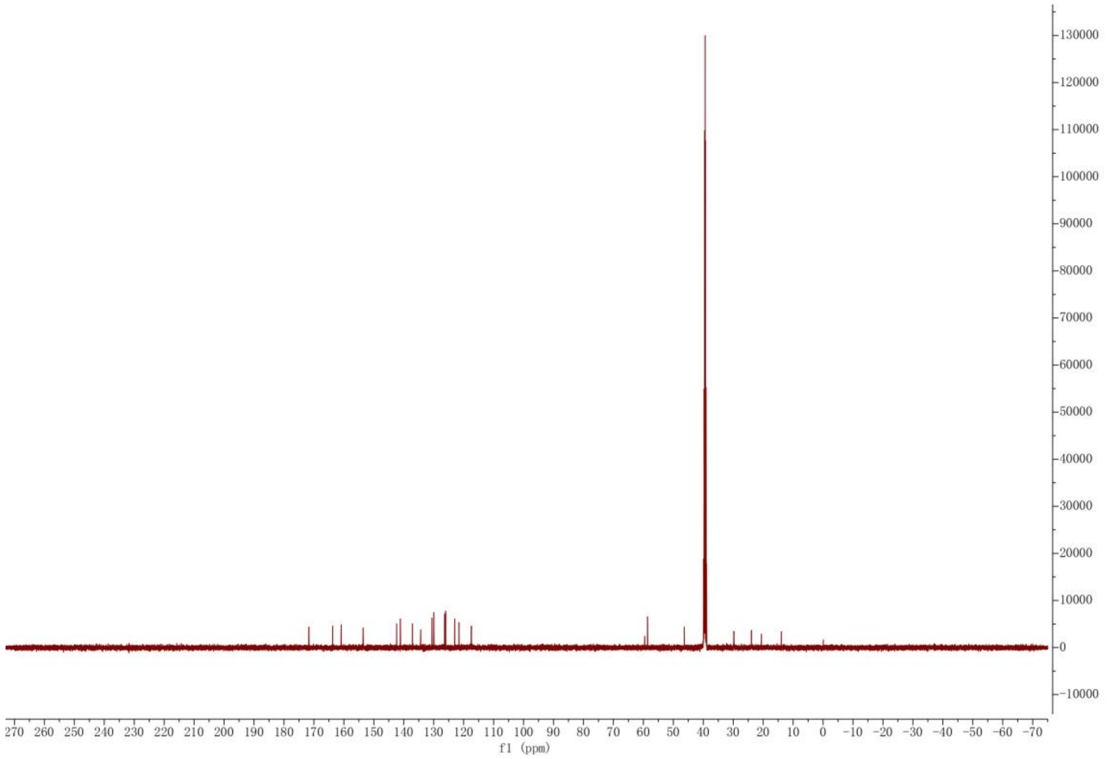

Figure S9. ^{13}C-NMR spectrum of compound **8i**.

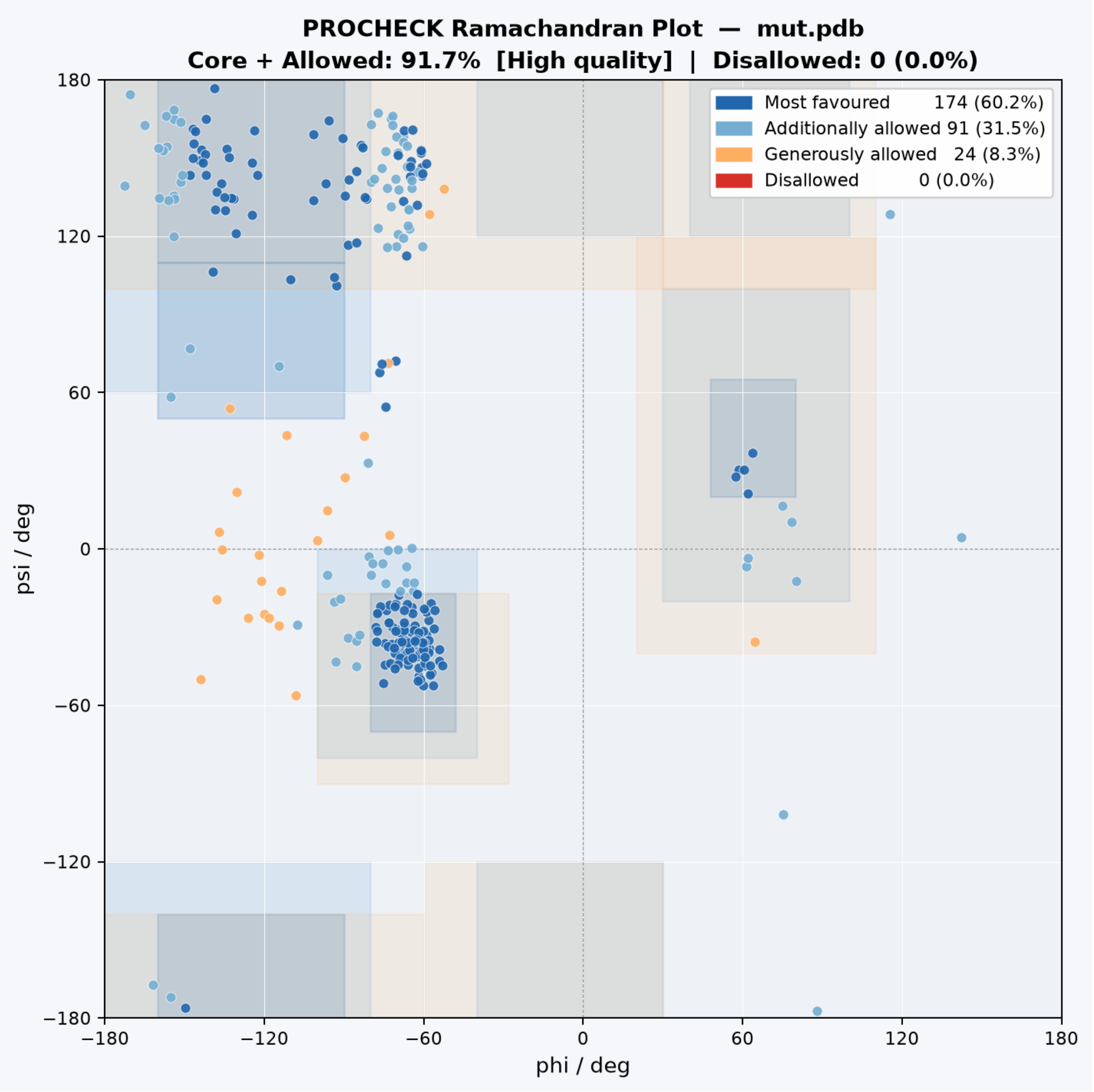

Figure S10. Ramachandran plot of the energy-minimized CDK4 mutant model.

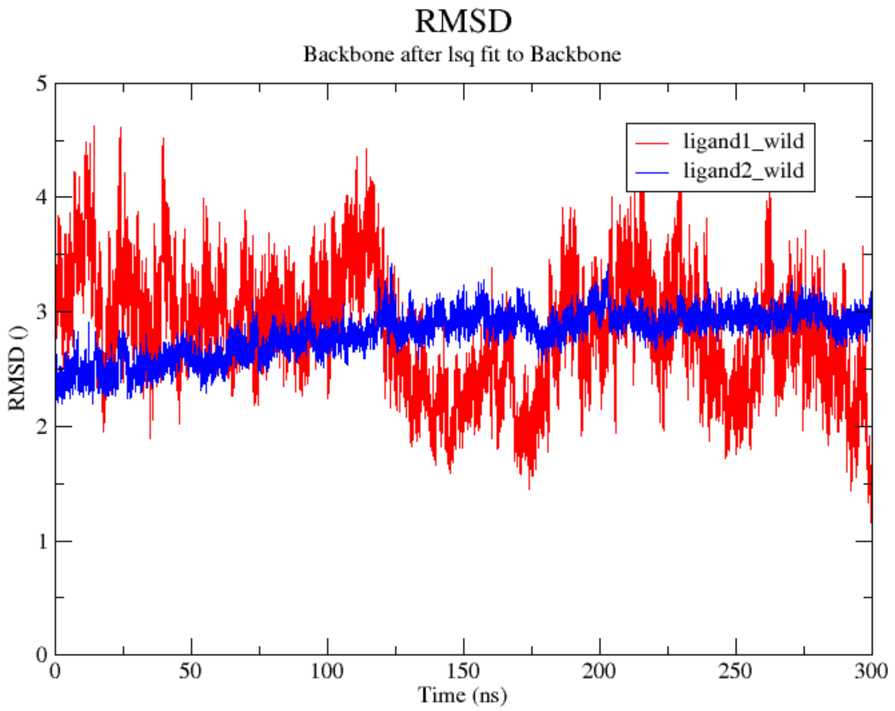

Figure S11: Chiral Isomer RMSD Comparison Plot

Molecular dynamics simulations demonstrated that the wild-type protein in complex with the Ligand1 enantiomer of compound 8i, the experimentally characterized lead compound in this study, exhibited more pronounced RMSD fluctuations than the corresponding Ligand2 complex, indicating enhanced conformational flexibility upon ligand binding. Such dynamic structural rearrangements are consistent with the conformational transitions commonly associated with allosteric regulation. By comparison, the Ligand2 enantiomer formed a more stable complex with the wild-type protein, as evidenced by the relatively small RMSD fluctuations throughout the simulation. These results suggest that the Ligand1 enantiomer of compound 8i is more likely to induce conformational changes in the wild-type protein and therefore may possess greater allosteric modulatory potential. However, additional computational analyses and experimental validation are warranted to confirm the proposed allosteric mechanism.

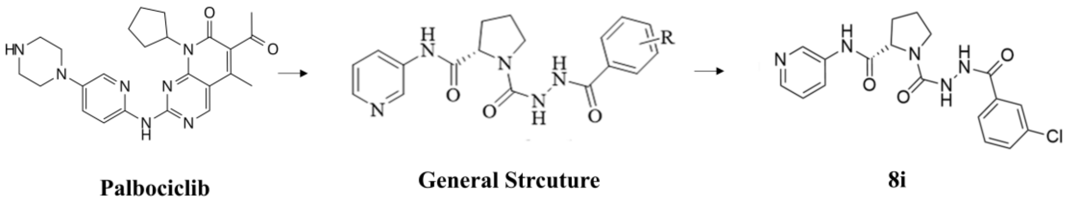

Figure S12: Design strategy of compound 8i. The chemical structures of the well-known CDK4/6 inhibitor Palbociclib and the starting general structure are shown in comparison with the newly designed target compound 8i.

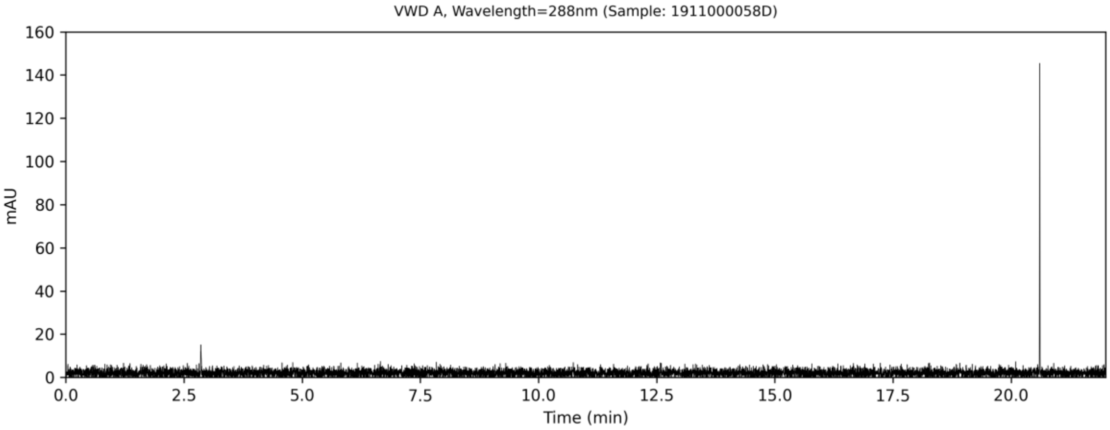

No.	RT (min)	Relative Area	FWHM (min)
**1**	2.859	0.89%	0.25
**2**	18.5	1.12%	0.28
**3**	19.8	0.67%	0.26
**4**	21.2	0.45%	0.24

Figure S13. Chromatogram of 8i

Table S1 Binding energy of wild-type and mutant protein-ligand complexes

name	Energy(KJ/mol) (mut-ligand)	Energy(KJ/mol) (wild-ligand)
VDWAALS	-2315.4383	-2346.8810
EEL	-19791.6748	-19862.1108
EGB	-2718.3304	-2680.2524
ESURF	107.3483	105.6920
G gas	-22107.1131	-22208.9918
G solv	-2610.9821	-2574.5605
TOTAL	-24718.0952	-24783.5523

Table S2. The water box information in the molecular dynamics research

		Wt	Mut
Water	N_{water}	14487	14187
Box size	x	51.7	51.2
(Å)	y	66.5	66.5
	z	48.2	48.0
N_{atoms}		48093	47184

Table S3. Enzymatic inhibitory activities of the compounds in our previous studies

Compound.	R	IC_{50}(nM)				Selectivity
		CDK6	CDK4	CDK2	CDK1	CDK2/CDK6
**7a**	H	10.29	6.23	970.55	1714.36	94.3
**7b**	2-F	2.07	3.28	392.87	227.89	189.8
**7c**	2-Cl	4.09	1.58	864.83	759.45	211.5
**7d**	2-NO_2	271.08	25.11	1371.25	12160.37	5.1
**7e**	2-Br	50.01	158.48	615.77	967.83	12.3
**7f**	2-CH_3	203.56	79.43	1589.73	1063.23	7.8
**7g**	2-OCH_3	12.29	6.31	253.78	389.80	20.7

**7h**	3-F	295.67	50.12	993.15	201.07	3.4
**7i**	3-Cl	7033.96	6309.57	365269.83	169587.23	51.9
**7j**	3-NO_2	895.57	199.52	79288.27	103268.19	88.5
**7k**	3-Br	1517.05	630.95	4229.58	1132.78	4.7
**7l**	3-CH_3	7675.38	6760.84	73694.37	96534.25	9.6
**7m**	3-OCH_3	3357.37	2511.43	59729.24	23632.83	17.8
**7n**	4-F	16.82	3.98	963.21	2710.94	57.3
**7o**	4-Cl	250.67	50.61	6547.53	4967.08	26.1
**7p**	4-NO_2	3.97	5.01	1025.11	2037.54	258.2
**7q**	4-Br	117.43	63.55	836.22	934.61	7.1
**7r**	4-CH_3	422.67	85.12	1027.38	1572.90	2.4
**7s**	4-OCH_3	62.08	34.67	1943.21	2206.71	31.3
**8c**	2-Cl	2781.57	3436.96	759.83	1095.58	0.3
**8p**	4-NO_2	1561.66	1732.13	364.03	178.25	0.2
**Abemaciclib**		10.05	4.33	511.30	647.32	51.1

Table S4. Chromatographic Parameters

Parameter	Value
Main Peak RT	20.605 min (1236.3 s)
Main Peak Area	96.73%
Peak Width (FWHM)	0.35 – 0.45 s (0.006 – 0.0075 min)
Impurity Peaks	3 – 6 (<1.5%)
Baseline Noise (RMS)	≈ 2 – 5 counts
Data Points	9909
